# DNA methylation-mediated suppression of endocytosis confers resistance to duck hepatitis A virus type 3

**DOI:** 10.1128/spectrum.00565-26

**Published:** 2026-06-15

**Authors:** Shaofei Li, Di Hu, Xiang Mei, Lionel Kinkpe, Shuaiqin Wang, Jie Wei, Shuisheng Hou, Yunsheng Zhang, Xia Wang

**Affiliations:** 1 College of Animal Science and Technology, Northwest A&F University, Yangling, Shaanxi, China; 2 State Key Laboratory of Animal Biotech Breeding, Institute of Animal Science, Chinese Academy of Agricultural Sciences, Beijing, China; 3 Inner Mongolia Key Laboratory of Duck Breeding, Chifeng, China; University of Florida College of Dentistry, Gainesville, Florida, USA

**Keywords:** DNA methylation, viral susceptibility, endocytosis, host–pathogen interaction, DHAV-3, duck

## Abstract

Host DNA methylation plays a crucial role in antiviral defense, yet its impact on viral susceptibility remains unclear. Using ducklings resistant (R) or susceptible (S) to duck hepatitis A virus type 3 (DHAV-3), we integrated whole-genome bisulfite and oxidative bisulfite sequencing with transcriptomics. We observed a distinct intragenic 5-methylcytosine (5mC) signature between the two lines, with endocytic pathway genes being hypomethylated in susceptible, while hypermethylated in resistant ducklings. Critically, the genebody 5mC level displayed a dominant negative correlation with gene expression, and functionally determined viral entry efficiency. Following infection, the methylation program further coordinated post-entry host responses, including immune, fatty acid metabolism, and autophagy pathways. This genebody methylation signature defines a regulatory framework governing DHAV-3 susceptibility, offering insights for antiviral strategies and disease-resistance breeding.

**IMPORTANCE:**

Duck hepatitis A virus type 3 (DHAV-3) causes severe mortality in ducklings, leading to substantial economic losses in poultry production. Understanding why some ducks resist infection while others succumb is critical for developing sustainable control strategies. Here, we discovered that a chemical “tag” on DNA—called methylation—acts as a natural switch that determines susceptibility. Resistant ducks carry higher methylation on genes controlling viral entry, effectively closing the door to the virus. Susceptible ducks lack this protective methylation, allowing viral access. Experimentally manipulating this switch directly altered infection efficiency, proving methylation is a functional controller of disease outcome. Our findings reveal that epigenetic variation, independent of genetic sequence, shapes host vulnerability. This opens new possibilities for breeding virus-resistant poultry through epigenetic markers, reducing reliance on vaccines and antiviral drugs. The work also provides a framework for understanding how animals defend against viral pathogens at the molecular level.

## INTRODUCTION

Viruses are structurally minimalist yet remarkably efficient pathogens that hijack host cellular machinery for replication and dissemination. Their infections impose a significant burden on human and animal health, ranging from acute, life-threatening diseases—such as those caused by SARS-CoV, MERS-CoV, Ebola virus, and HIV (1–4)—to chronic conditions that promote long-term pathology, including cirrhosis and hepatocellular carcinoma associated with HBV and HCV (5). Moreover, viral infections are implicated in neurological disorders and other systemic complications (6–8). This persistent threat underscores the critical need for effective antiviral strategies. While vaccines and antiviral drugs remain essential tools, the rapid evolution of viruses through mutation and recombination enables immune evasion and complicates long-term control (9). Consequently, exploring novel host-centered mechanisms of defense represents a promising avenue for intervention.

DNA methylation, a fundamental epigenetic modification primarily involving the addition of a methyl group to cytosine within CpG dinucleotides (forming 5-methylcytosine, 5mC) (10), serves as a key regulator of gene expression (11). 5mC generally promotes a transcriptionally repressive chromatin state (12, 13). This mark can be actively modified through oxidation by ten-eleven translocation enzymes, yielding 5-hydroxymethylcytosine (5hmC) and other intermediates in a dynamic demethylation pathway (14, 15). Both 5mC and 5hmC play crucial roles in fine-tuning transcriptional programs (16, 17). Dysregulation of DNA methylation is a hallmark of various diseases, including cancer and immune disorders, and is increasingly recognized as a pivotal factor in viral pathogenesis (18–22). Viruses actively manipulate the host epigenetic landscape to subvert antiviral defenses—for instance, by promoting methylation-mediated silencing of the complement system (23), interferon-stimulated genes, or pattern-recognition receptors (24–28). Conversely, the host can employ DNA methylation as a defensive mechanism, with specific methylation signatures correlating with resistance to pathogens (29–31). Notably, susceptibility to several viruses, including SARS-CoV-2 (32, 33), Marek’s disease virus (29), and avian leukosis virus (34), has been linked to distinct host DNA methylation patterns, highlighting its potential role in determining infection outcomes.

To dissect the mechanism by which DNA methylation influences viral susceptibility, we employed a robust avian model: ducklings with genetically determined resistance or susceptibility to duck hepatitis A virus type 3 (DHAV-3), a highly pathogenic picornavirus causing severe economic losses in waterfowl production (35, 36). In our previous work, eight successive generations of artificial selection yielded resistant ducklings with ~90% survival post-DHAV-3 infection, alongside susceptible ducklings with ~70% mortality (37, 38). Using oxidative and conventional whole-genome bisulfite sequencing (oxWGBS and WGBS), we profiled 5mC and 5hmC landscapes in resistant and susceptible ducklings before and after DHAV-3 infection. By integrating these epigenomic profiles with transcriptomic data and functional validation, we aimed to identify the specific methylation-regulated pathways that govern host susceptibility. This study provides novel insights into the epigenetic basis of host–pathogen interactions and may inform the development of epigenetic-based antiviral strategies and breeding programs for disease-resistant poultry.

## RESULTS

### Pathological analysis revealed more severe liver damage in susceptible ducklings during DHAV-3 infection

**Fig 1:**
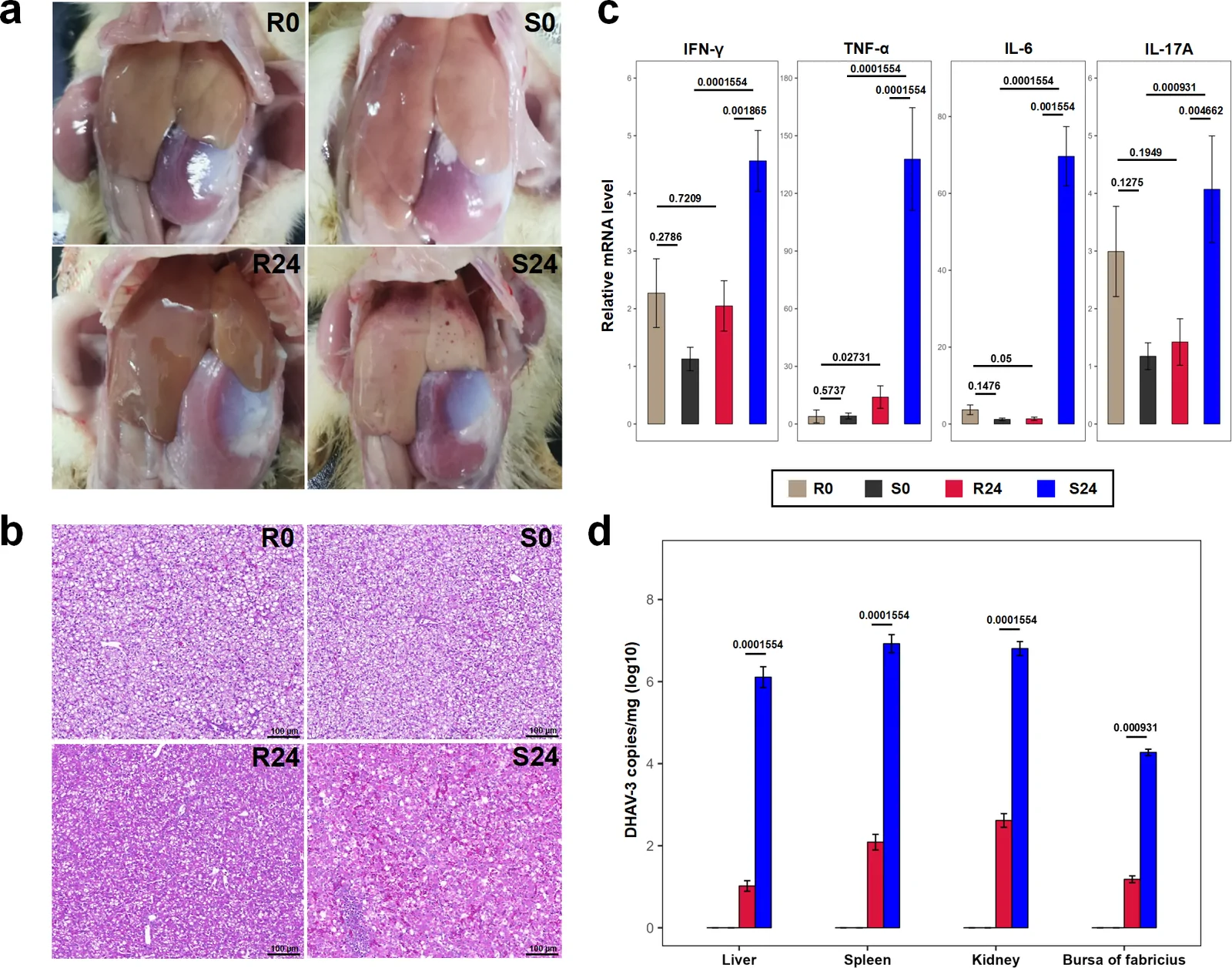
Pathological traits of resistant (R) and susceptible (S) ducklings during DHAV-3 infection. (**a**) Representative gross liver morphology from uninfected (R0, S0) and DHAV-3-infected (R24, S24) ducklings at 24 hpi. (**b**) Representative hematoxylin and eosin staining of liver sections from each group. (**c**) Relative mRNA expression levels of pro-inflammatory cytokines (*IFN-γ*, *TNF-α*, *IL-6*, *IL-17A*) in the liver at 0 and 24 hpi. Expression levels were normalized to GAPDH and are presented as mean ± SEM (*n* = 3). (**d**) Viral loads in the liver, spleen, kidney, and bursa of Fabricius at 24 hpi. Viral RNA copies were quantified by qPCR and the DHAV-3 standard curve. Statistical significance was determined by a two-tailed Wilcoxon rank-sum test.

To assess infection-associated pathology, livers from resistant (R) and susceptible (S) ducklings were collected before (0 hpi) and after (24 hpi) DHAV-3 challenge. Macroscopically, livers from uninfected controls (R0, S0) appeared normal, with smooth capsules and a uniform reddish-brown parenchyma (Fig. 1a). In stark contrast, livers from infected susceptible ducklings (S24) exhibited marked pallor with multifocal petechial hemorrhages. Histopathological analysis revealed extensive hepatocellular necrosis, erythrocyte pooling, and pronounced inflammatory infiltrates in S24 livers—characteristic findings of severe duck viral hepatitis (Fig. 1b). This severe tissue damage was associated with a significant upregulation of pro-inflammatory cytokines (*IFN-γ*, *TNF-α*, *IL-6*, *IL-17A*) in the S24 group compared to all others (Fig. 1c). Furthermore, viral loads in the liver, spleen, kidney, and bursa of Fabricius were substantially higher in S24 than in R24 (Fig. 1d), indicating uncontrolled viral infection in S-ducklings.

### Genome-wide methylation and hydroxymethylation profiles in resistant and susceptible ducklings

To investigate epigenetic differences underlying ducklings’ susceptibility to DHAV-3, we performed oxidative bisulfite (oxWGBS) and conventional bisulfite sequencing (WGBS) on liver samples from R and S ducklings at 0 and 24 hpi. Each library yielded ~130 million clean reads, with Q20 > 98%, Q30 > 94%, mapping rates > 89%, and bisulfite conversion rates ~99.5% (Table 1).

**TABLE 1:** Summary of WGBS and oxWGBS libraries

	Sample	Raw reads	Clean reads	Q20 (%)	Q30 (%)	Map rate (%)	BS_conversion (%)
oxWGBS	R0_1	131,229,501	129,876,913	98.29	95.05	89.65	99.48
	R0_2	134,122,443	132,727,720	98.16	94.65	89.90	99.46
	R0_3	139,694,154	138,376,226	98.25	94.92	88.70	99.40
	S0_1	121,713,242	120,570,423	98.35	95.31	90.05	99.47
	S0_2	126,237,865	125,071,311	98.39	95.38	89.20	99.43
	S0_3	122,140,792	120,934,674	98.05	94.29	89.65	99.39
	R24_1	130,729,230	129,351,375	98.45	95.57	88.20	99.46
	R24_2	127,098,995	125,640,083	98.25	94.82	89.85	99.51
	R24_3	122,863,754	121,482,231	98.22	94.73	89.80	99.47
	S24_1	121,874,840	120,667,108	98.20	94.69	89.20	99.45
	S24_2	158,368,663	156,667,877	98.33	95.15	89.10	99.49
	S24_3	130,343,014	129,002,431	98.32	95.10	89.70	99.61
WGBS	R0_1	138,539,877	136,976,346	98.37	95.38	90.20	99.40
	R0_2	127,638,162	126,086,194	98.09	94.39	89.35	99.38
	R0_3	125,708,622	124,204,891	98.38	95.36	89.45	99.40
	S0_1	124,688,873	123,356,880	98.33	95.23	89.55	99.40
	S0_2	134,869,946	133,366,793	98.23	94.74	90.25	99.40
	S0_3	134,283,586	132,528,121	98.42	95.37	90.30	99.40
	R24_1	137,416,225	135,428,318	98.38	95.32	90.45	99.40
	R24_2	146,174,140	144,407,169	97.57	92.97	90.55	99.36
	R24_3	126,390,824	125,049,185	98.18	94.70	89.35	99.38
	S24_1	127,556,497	126,167,001	98.14	94.61	89.70	99.38
	S24_2	148,777,591	147,275,695	98.03	94.25	89.55	99.37
	S24_3	139,060,721	137,596,393	98.09	94.46	89.90	99.36

**Fig 2:**
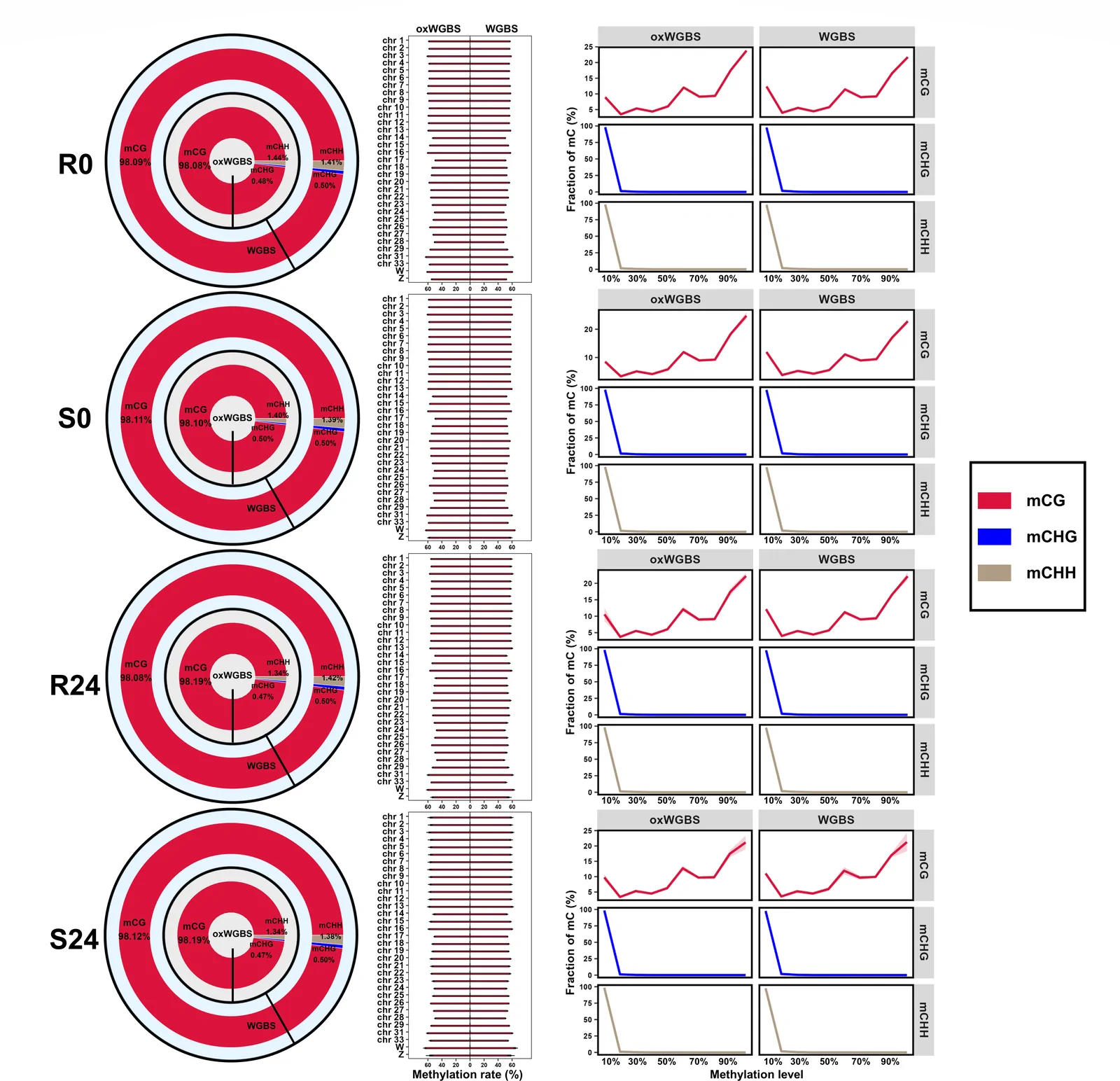
Characteristics of oxWGBS and WGBS libraries from uninfected and DHAV3-infected ducklings. The pie charts showed the percentages of mCG, mCHG, and mCHH at methylcytosines. The methylation levels for each chromosome were displayed as bar charts. The line charts reflected the distribution of methylation for all groups.

Global cytosine methylation (mC) levels averaged 2.96% (oxWGBS) and 3.21% (WGBS). Methylation in CG contexts (mCG) accounted for >98% of all mC sites, with an average methylation level (ML) of 57.34%. In contrast, methylation in CHG and CHH contexts was minimal (<0.07%) (Table S1; Fig. 2). Approximately half of all mCG sites were highly methylated (ML > 70%), whereas mCHG and mCHH sites showed low methylation (ML < 20%) (Fig. 2). Given the predominance of mCG, subsequent analyses focused on this context to capture valuable insights.

**Fig 3:**
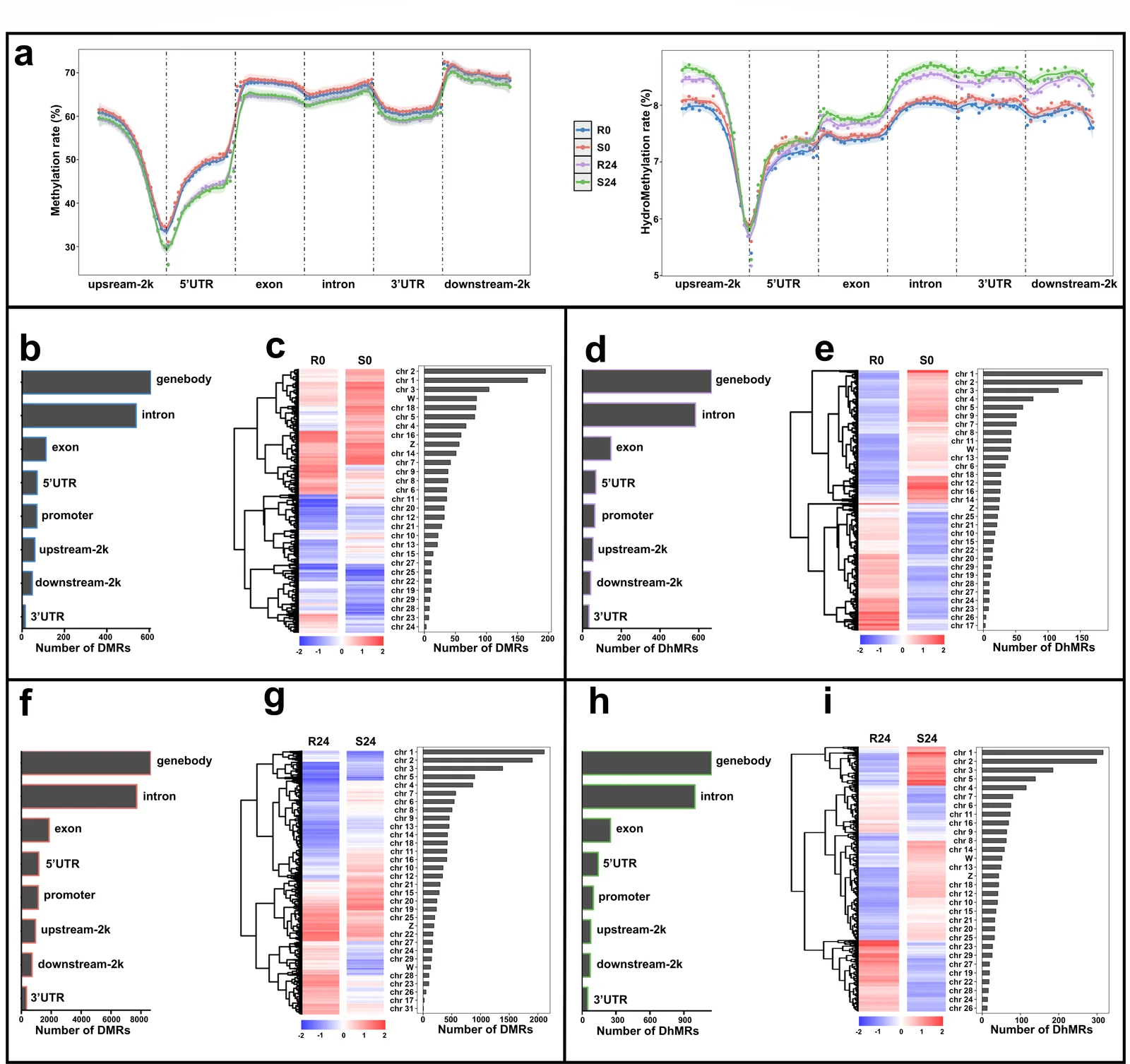
Global methylation and hydroxymethylation profiles of resistant and susceptible ducklings. (**a**) Methylation rate and hydroxymethylation rate across different gene elements, including upstream 2k, 5′UTR, exons, introns, 3′UTR, and downstream 2k. (**b**) Distribution of differentially methylated regions (DMRs) between R0 and S0 in different gene elements. (**c**) Distribution of DMRs between R0 and S0 on chromosomes. (**d**) Distribution of differentially hydroxymethylated regions (DhMRs) between R0 and S0 in different gene elements. (**e**) Distribution of DhMRs between R0 and S0 on chromosomes. (**f**) Distribution of DMRs between R24 and S24 in different gene elements. (**g**) Distribution of DMRs between R24 and S24 on chromosomes. (**h**) Distribution of DhMRs between R24 and S24 in different gene elements. (**i**) Distribution of DhMRs between R24 and S24 on chromosomes.

We next examined 5mC and 5hmC distribution across functional genomic regions. Following infection, both R24 and S24 showed decreased 5mC levels, particularly near transcription start sites (TSS), 5′UTRs, and exons, whereas 5hmC levels increased relative to uninfected controls (Fig. 3a).

### DHAV-3 infection massively expands DNA methylation divergence between resistant and susceptible ducklings

**Fig 4:**
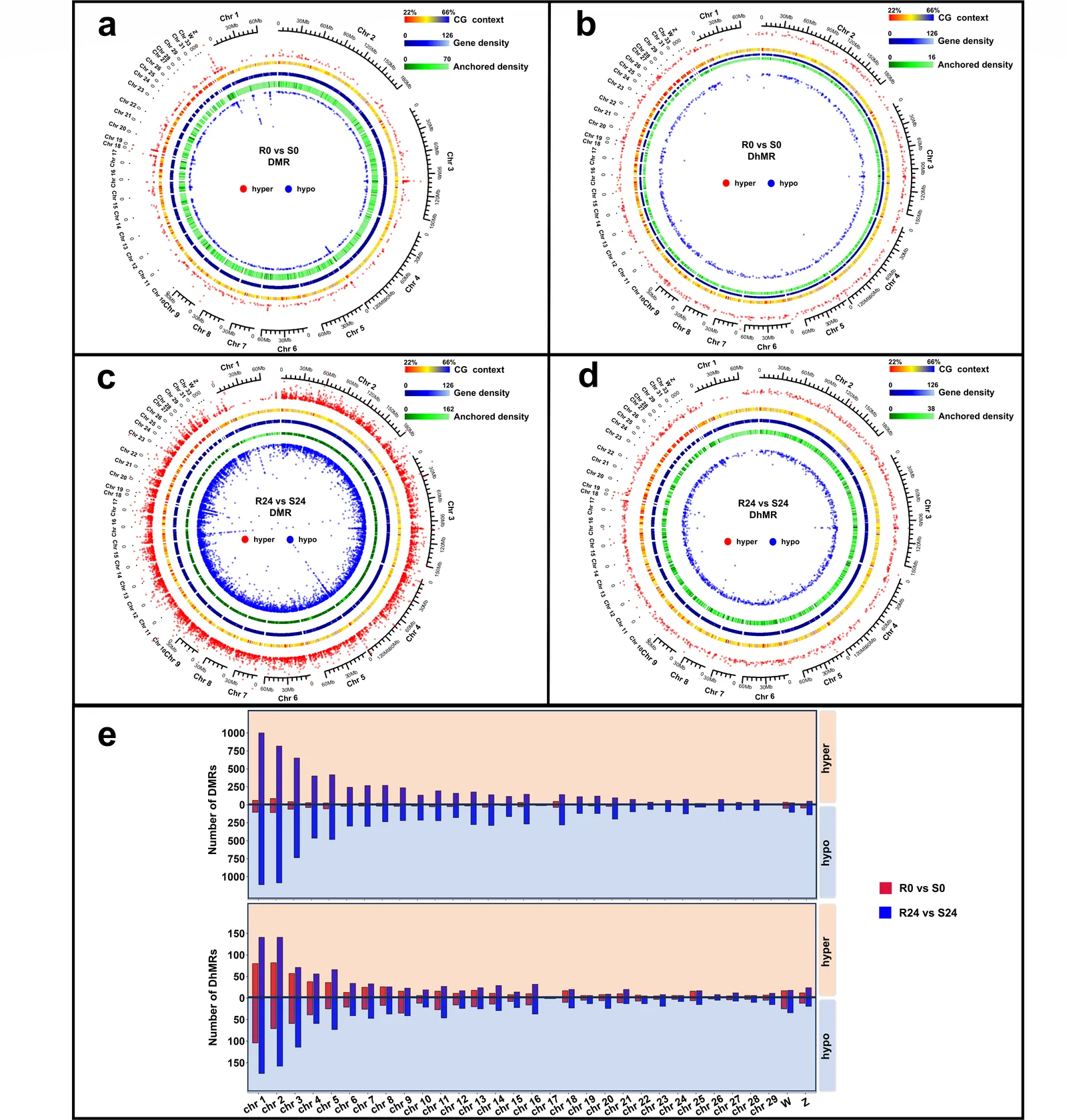
Distribution of hypermethylated (hyper) and hypomethylated (hypo) DMRs and DhMRs. (**a**) Distribution of hyper- and hypo-DMRs between R0 and S0. (**b**) Distribution of hyper- and hypo-DhMRs between R0 and S0. (**c**) Distribution of hyper- and hypo-DMRs between R24 and S24. (**d**) Distribution of hyper- and hypo-DhMRs between R24 and S24. Red dots indicate hyper-DMRs/DhMRs, blue dots represent hypo-DMRs/DhMRs. (**e**) The number of hyper- and hypo-DMRs/DhMRs on each chromosome at 0 and 24 hpi.

Comparative analysis identified 1,359 differentially methylated regions (DMRs) and 1,192 differentially hydroxymethylated regions (DhMRs) between R0 and S0. After DHAV-3 infection, DMRs expanded dramatically to 14,537 between the R24 and S24, and DhMRs increased to 2,092 (Tables S2 and S3). Most DMRs/DhMRs were <400 bp in length (Fig. S1). Genomic and chromosomal distributions of DMRs and DhMRs were similar: both were enriched in genebody (predominantly introns) and concentrated on the largest three autosomes (Fig. 3b through i). While the numbers of hyper- and hypo-hydroxymethylated regions (hyper-/hypo-DhMRs) remained similar before and after infection, both hyper- and hypo-methylated regions (hyper-/hypo-DMRs) increased markedly at 24 hpi (Fig. 4a through d). The top three autosomes contained an average of 64 hyper-DMRs and 91 hypo-DMRs at 0 hpi, and 823 hyper-DMRs and 977 hypo-DMRs at 24 hpi (Fig. 4e).

### Genebody 5mC levels are the primary epigenetic determinant of gene expression changes

**Fig 5:**
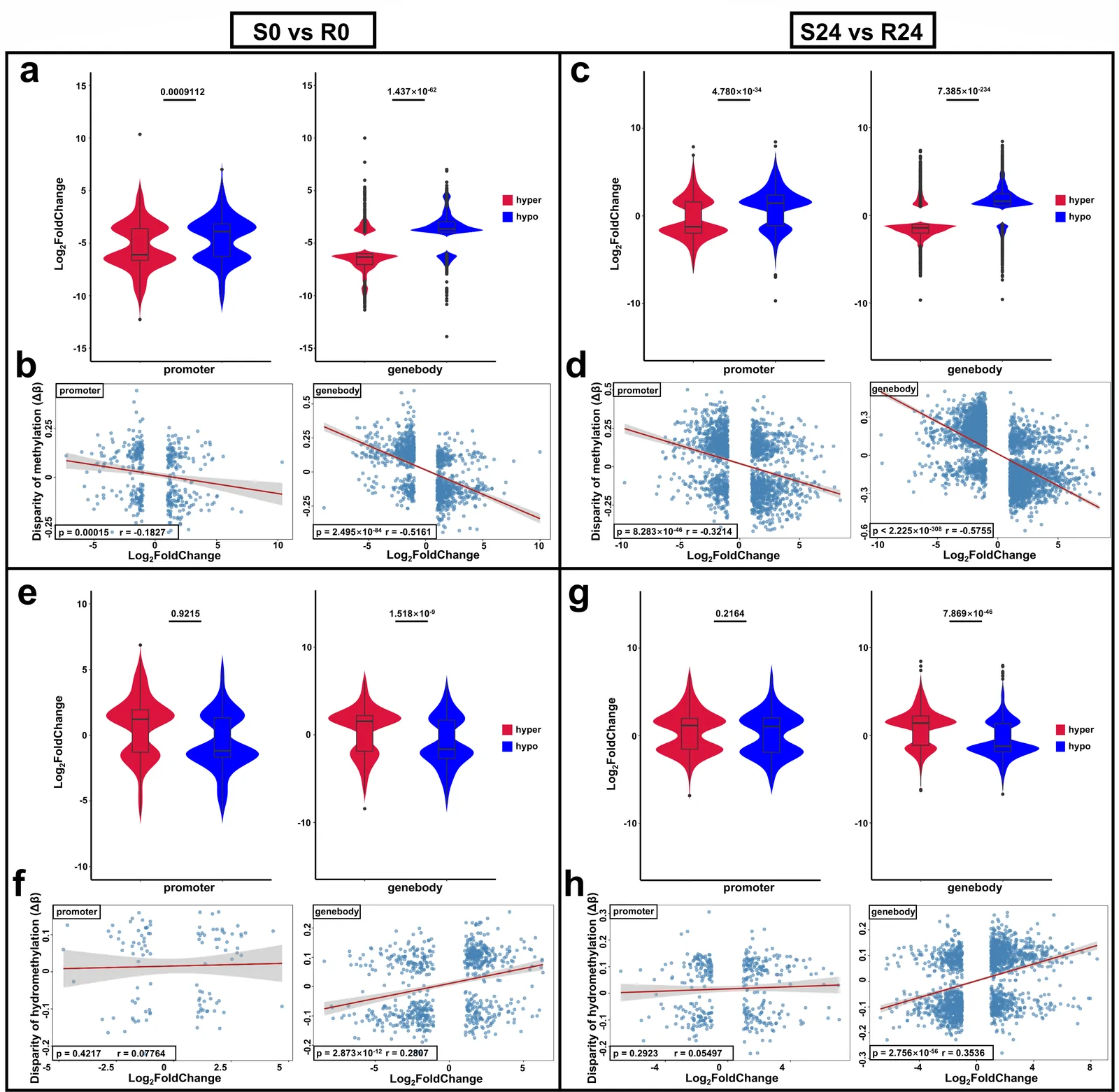
Integration analysis of DNA methylation and hydroxymethylation with gene expression between R- and S-ducklings. The log2FoldChange values of the expression levels of hyper- and hypo-DMR-anchored genes (DMGs) at 0 hpi (**a**) and 24 hpi (**c**). Statistical significance was determined by a two-tailed Wilcoxon rank-sum test. Spearman correlation analysis of the mRNA expression changes and methylation levels of DMGs at 0 hpi (**b**) and 24 hpi (**d**). The log2FoldChange values of the expression levels of hyper- and hypo-DhMR-anchored genes (DhMGs) at 0 hpi (**e**) and 24 hpi (**g**). Spearman correlation analysis of the mRNA expression changes and methylation levels of DMGs at 0 hpi (**f**) and 24 hpi (**h**). Hyper, hypermethylated; hypo, hypomethylated.

We analyzed genes anchored by DMRs (DMGs) and DhMRs (DhMGs) to assess the relationship between methylation and transcription. A strong negative correlation was observed between 5mC levels (Δβ) and gene expression changes (log₂FC) for DMGs in both promoter and genebody regions at 0 hpi (promoter: *P* = 0.00015, r = −0.1827; genebody: *P* = 2.495 × 10^−84^, r = −0.5161) and 24 hpi (promoter: *P* = 8.283 × 10^−46^, r = −0.3214; genebody: *P* < 2.225 × 10^−304^, r = −0.5755) (Fig. 5a through d). In contrast, promoter DhMGs showed no correlation with expression (0 hpi: *P* = 0.4217, r = 0.07764; 24 hpi: *P* = 0.2923, r = 0.0597), while genebody DhMGs exhibited a positive correlation—opposite to DMGs (0 hpi: *P* = 2.873 × 10^−12^, r = 0.2807; 24 hpi: *P* = 2.756 × 10^−56^, r = 0.3536) (Fig. 5e through h). Similar trends were observed between uninfected and infected groups (R24 vs R0; S24 vs S0) (Fig. S2).

**Fig 6:**
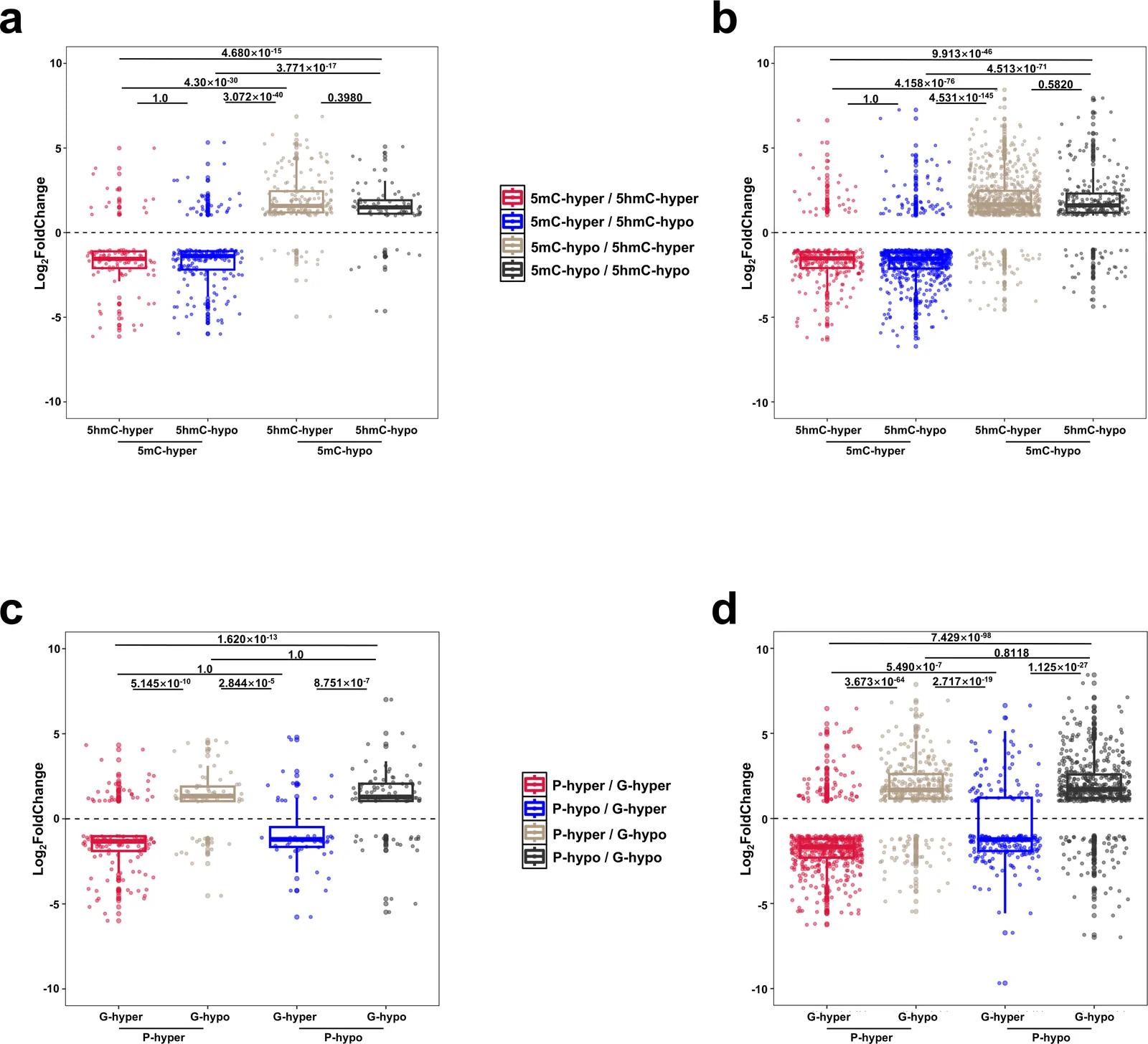
The relationship between 5mC, 5hmC, and gene expression. Grouped gene expression changes of DMGs and DhMGs at 0 hpi (**a**) and 24 hpi (**b**). Grouped gene expression changes of DMGs in promoter and genebody regions at 0 hpi (**c**) and 24 hpi (**d**). Hyper, hypermethylated; hypo, hypomethylated; P, promoter; G, genebody.

To dissect the individual contributions of 5mC and 5hmC modifications in promoter and genebody, DMGs and DhMGs were classified as 5mC-hyper (hypermethylated-DMG), 5mC-hypo (hypomethylated-DMG), 5hmC-hyper (hypermethylated-DhMG), or 5hmC-hypo (hypomethylated-DhMG). Within the same 5hmC group (hyper/hypo), 5mC-hyper genes showed significantly lower log₂FC than 5mC-hypo genes at both time points (Fig. 6a and b). Conversely, within the same 5mC group, 5hmC-high and 5hmC-low genes showed comparable log₂FC, indicating that 5mC—not 5hmC—drives expression changes.

We further examined DMGs in both promoter and genebody. At 0 hpi, G-hypo genes (hypomethylated-DMGs in genebody) showed higher log₂FC than G-hyper genes (hypermethylated-DMGs in genebody) within the same promoter group, whereas promoter methylation had no significant effect (Fig. 6c). At 24 hpi, although promoter methylation showed some influence within the G-hyper group, overall expression remained suppressed (median log₂FC < −1), suggesting that high genebody 5mC dominates the regulatory outcome (Fig. 6d).

### Genebody 5mC modifications regulate endocytosis, fatty acid metabolism, autophagy, and immune responses during DHAV-3 infection

**Fig 7:**
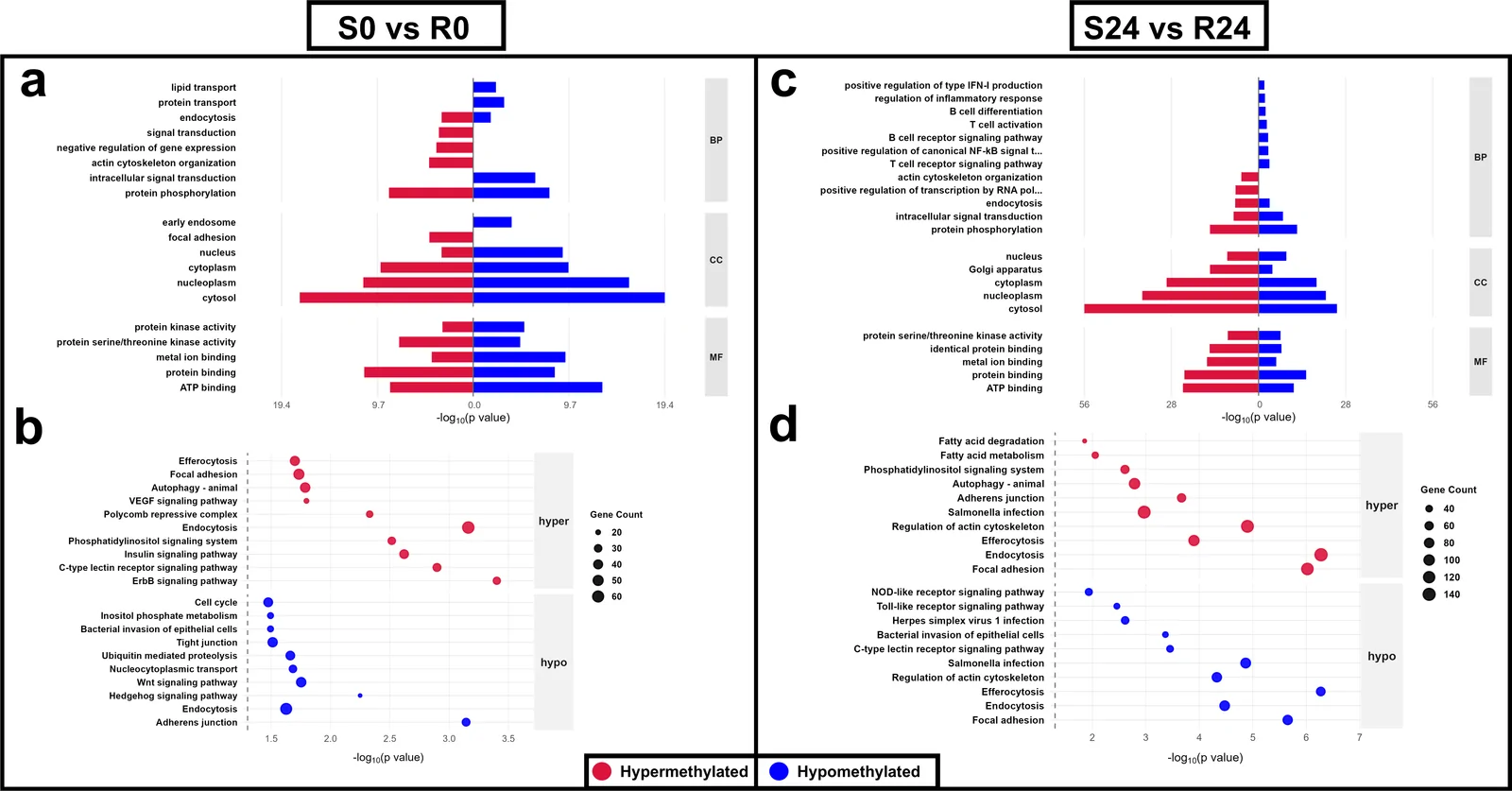
Functional enrichment analysis of 5mC-modified genes in the genebody. Gene Ontology (GO) enrichment analysis of hyper- and hypo-DMGs between S- and R-ducklings at 0 hpi (**a**) and 24 hpi (**c**). Functional clusters of Kyoto Encyclopedia of Genes and Genomes (KEGG) for hyper- and hypo-DMGs at 0 hpi (**b**) and 24 hpi (**d**).

Having established that genebody 5mC is the dominant regulatory layer, we next investigated the biological pathways associated with these methylation changes. Functional enrichment of DMGs in genebody regions revealed distinct pathways associated with ducklings’ susceptibility to DHAV-3. In uninfected ducklings, S0 hyper-DMGs were enriched in pathways, including signal transduction, negative regulation of gene expression, focal adhesion, autophagy, and efferocytosis, while hypo-DMGs were mapped to lipid transport, protein transport, early endosomes, and cell junctions (Fig. 7a and b). In infected ducklings, S24 hyper-DMGs were enriched in actin cytoskeleton regulation, RNA polymerase II-mediated transcription, focal adhesion, efferocytosis, autophagy, and fatty acid metabolism/degradation, while hypo-DMGs were concentrated in immune pathways, including T/B cell activation, IFN-I production, and NLR/TLR signaling (Fig. 7c and d). Detailed enriched items are listed in Tables S4 and S5.

**Fig 8:**
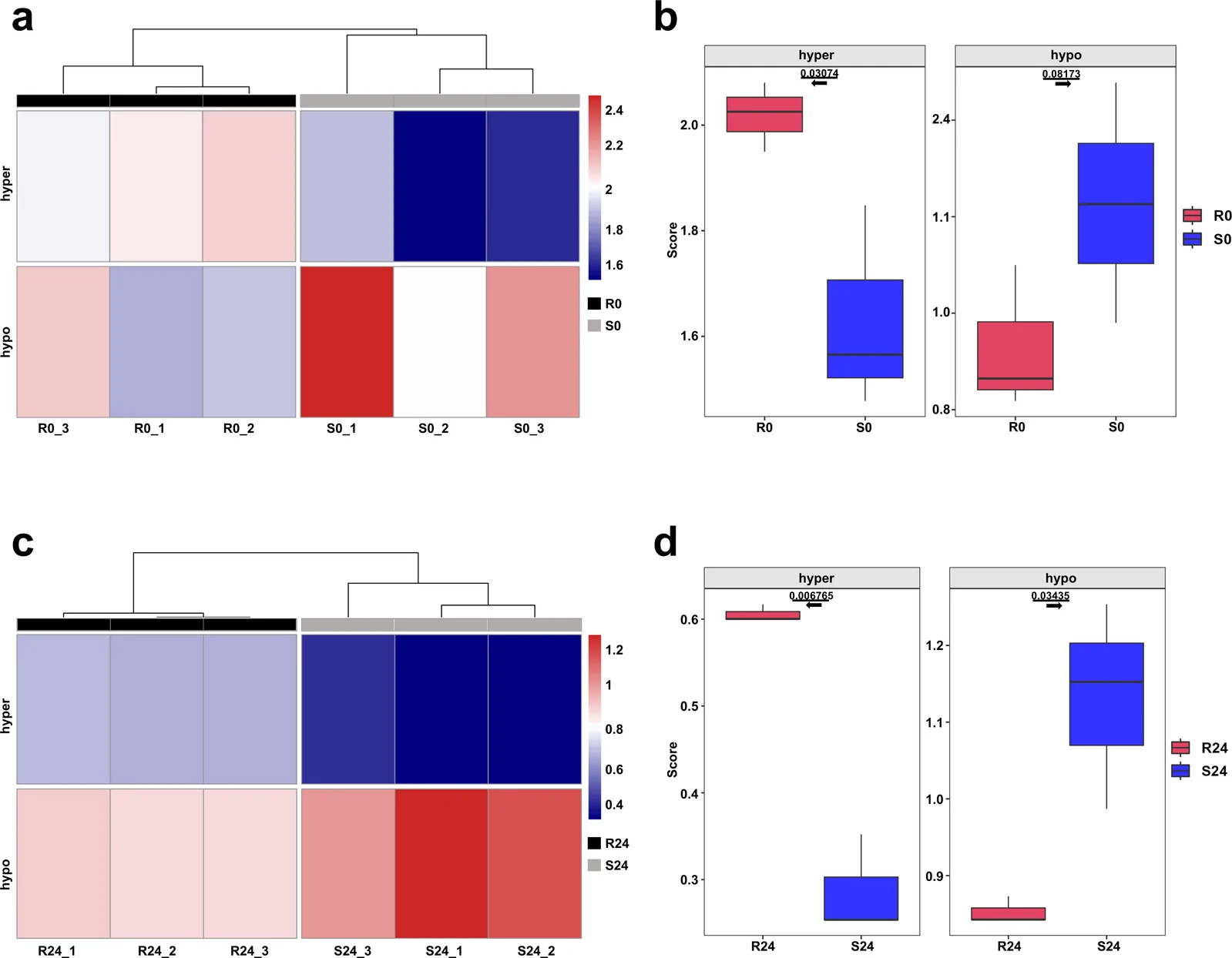
ssGSEA analysis of the gene set related to endocytosis. Enrichment heatmap (**a**) and enrichment scores (**b**) of hyper- and hypo-DMGs associated with endocytosis between S0 and R0. Enrichment heatmap (**c**) and enrichment scores (**d**) of hyper- and hypo-DMGs associated with endocytosis between S24 and R24.

Notably, endocytosis was enriched in both hyper- and hypo-DMGs at 0 and 24 hpi. S-ducklings predominantly harbored hypo-DMGs in endocytosis-related genes, whereas R-ducklings showed hyper-DMGs (Fig. S3). Single-sample gene set enrichment analysis (ssGSEA) confirmed lower endocytosis pathway enrichment of hyper-DMGs in S0 and S24, and higher enrichment of hypo-DMGs in S24, suggesting that reduced 5mC in S-ducklings augments endocytosis gene expression (Fig. 8). Furthermore, autophagy pathways were hypermethylated in both ducklings during viral infection, while fatty acid metabolism and immune pathways were exclusively hypermethylated in S-ducklings (Fig. S3).

### DNA methylation suppression enhances DHAV-3 endocytosis

**Fig 9:**
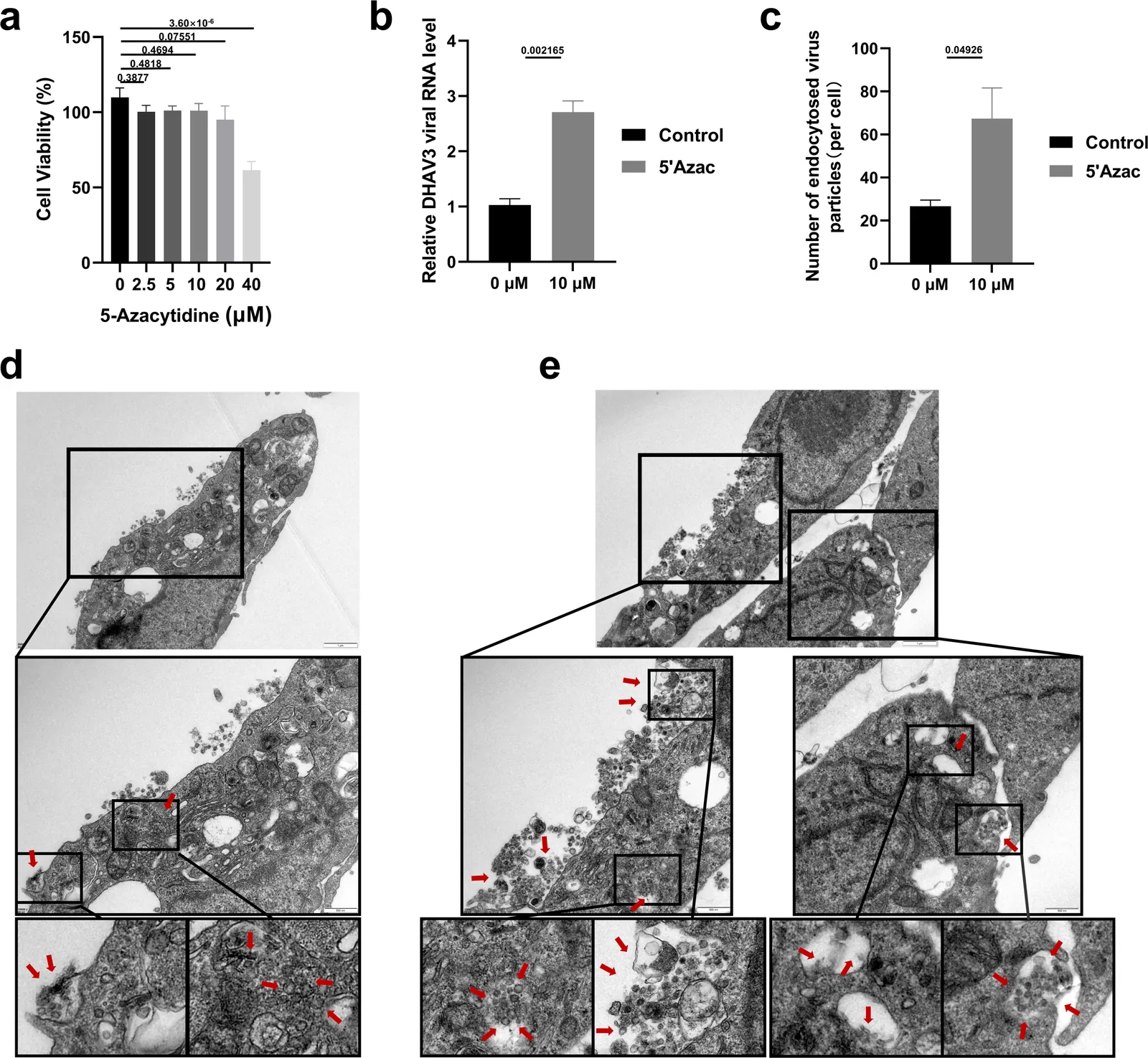
The effect of 5mC modification on the endocytosis of DHAV3. (**a**) The CCK-8 assay of 5′Azac. (**b**) Viral RNA loads in 5′Azac-treated cells and controls following DHAV3 infection. (**c**) The number of endocytosed DHAV3 viral particles was determined by transmission electron microscopy (TEM). TEM results of cells treated with 1/10,000 DMSO (**d**) and 10 μM 5′Azac (**e**) following DHAV3 infection. In each panel, the magnified regions are different areas taken from the same original TEM field, selected to illustrate viral entry events. The endocytosed virions are indicated by red arrows.

To functionally validate the role of 5mC, we treated primary duck hepatocytes with the DNA methyltransferase inhibitor 5-Azacytidine (5′AzaC). At non-cytotoxic concentrations, 5′AzaC significantly increased intracellular DHAV-3 RNA levels (Fig. 9a and b). Transmission electron microscopy revealed a higher number of viral particles within endocytic vesicles in 5′AzaC-treated cells compared to controls (Fig. 9c). Characteristic membrane invaginations and vesicular internalization of enveloped virions were evident at sites of viral attachment (Fig. 9d and e).

### Modulation of fatty acid metabolism and autophagy influences DHAV-3 replication

**Fig 10:**
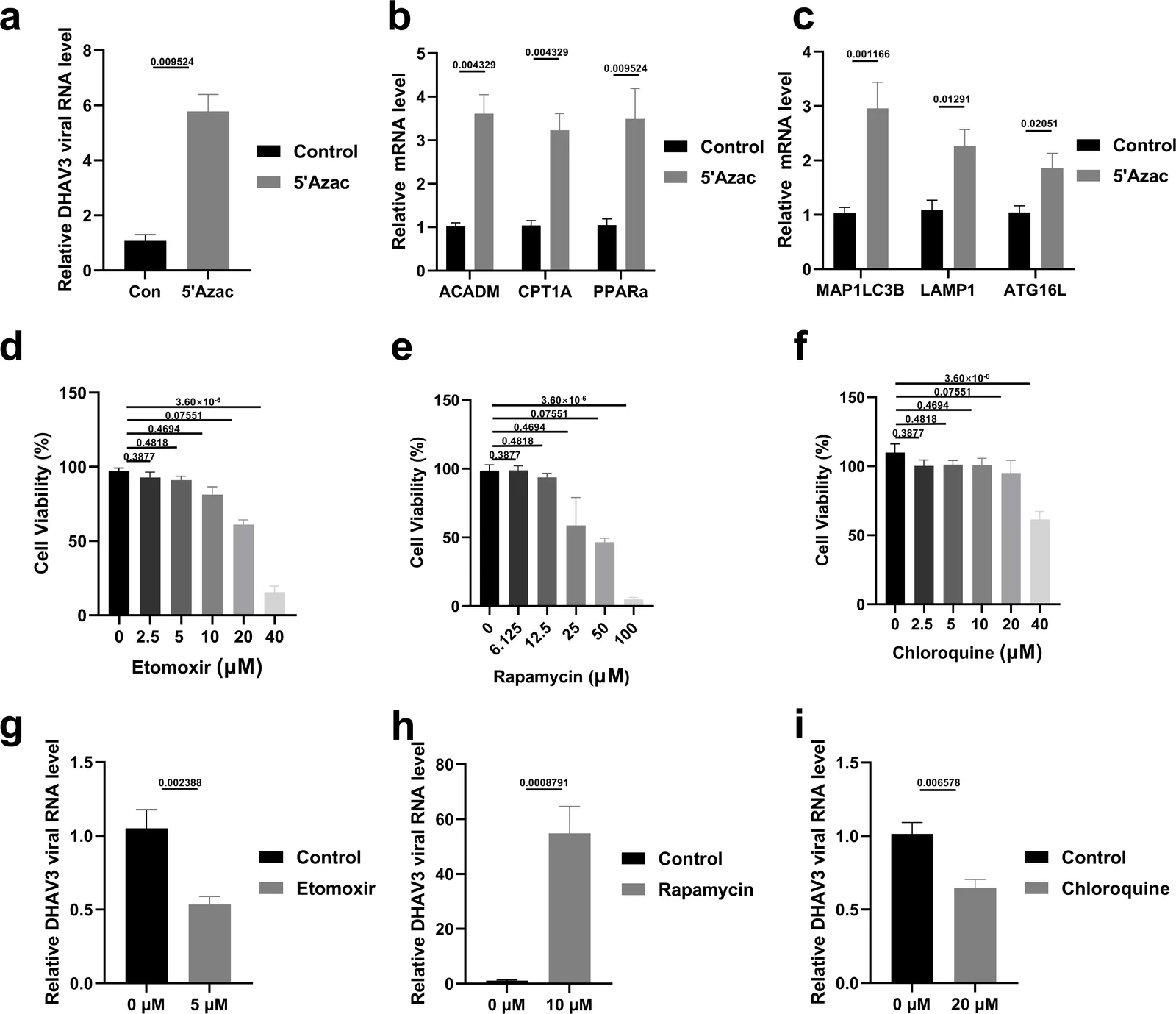
The effects of fatty acid metabolism and autophagy on the replication of DHAV3. (**a**) Viral RNA levels of DHAV3 in the 5′Azac-treated cells and the control group at 24 hpi, measured by qPCR and normalized to *GAPDH*. (**b**) The mRNA levels of fatty acid metabolism-related genes *ACADM*, *CPT1A*, and *PPAR*α in 5′Azac-treated cells and controls at 24 hpi. (**c**) The mRNA levels of autophagy-related genes *MAP1LC3B, LAMP1,* and *ATG16L* in 5′Azac-treated cells and controls at 24 hpi. The CCK-8 assay results of etomoxir (**d**), rapamycin (**e**), and chloroquine (**f**). Viral RNA levels of DHAV3 in cells treated with etomoxir (**g**), rapamycin (**h**), chloroquine (**i**), and controls were measured at 24 hpi.

Given the enrichment of fatty acid metabolism and autophagy pathways among hyper-DMGs, we pharmacologically perturbed these processes. 5′AzaC treatment increased not only viral load but also the expression of fatty acid metabolism genes (*ACADM*, *CPT1A*, *PPAR*α) and autophagy markers (*MAP1LC3B*, *LAMP1*, *ATG16L*) during infection (Fig. 10a through c). Inhibition of fatty acid metabolism with etomoxir reduced viral RNA levels at 24 hpi (Fig. 10d and g). Similarly, autophagy induction with rapamycin enhanced viral replication, whereas inhibition with chloroquine suppressed it (Fig. 10e, f, h, and i). These results suggest that host resistance is partly mediated by 5mC-mediated suppression of fatty acid metabolism and autophagy, pathways that are otherwise exploited by DHAV-3.

## DISCUSSION

DNA methylation is increasingly recognized as a key epigenetic regulator of host–pathogen interactions across diverse species, playing a conserved yet complex role in viral pathogenesis and host susceptibility (20, 21, 32, 39–42). In this study, we employed integrated WGBS, oxWGBS, and transcriptomics to dissect the DNA methylation landscapes underlying differential susceptibility to DHAV-3 in ducks. Our analysis reveals that an intragenic methylation signature, rather than promoter methylation, distinguishes resistant from susceptible ducklings and governs viral entry and downstream host responses, providing mechanistic insights into epigenetic regulation of antiviral defense.

In vertebrate genomes, DNA methylation occurs predominantly in a CG context, with non-CG methylation (mCHG, mCHH) being relatively rare and tissue-specific, such as the brain and embryonic stem cells (43, 44). CG dinucleotides often cluster into CpG islands, which associate with promoters to regulate transcription (45)—typically remaining unmethylated, while non-island CG sites are more prone to methylation, resulting in lower promoter ML relative to other genomic elements (46). We found that the duck methylome follows this conserved pattern, with mCG constituting >98% of all modified sites and sharp reductions in ML upstream of TSS. Following DHAV-3 infection, we observed a global reduction in 5mC (particularly near TSS) and an increase in 5hmC. This shift suggests an active, infection-driven remodeling of the hepatic epigenome, potentially reflecting a viral strategy to dysregulate host transcription while also indicating a host epigenetic response to infection. Such global methylation changes have been reported in other viral infections, where they can either exacerbate disease—such as hypomethylation of inflammatory genes correlates with excessive cytokine production in severe COVID-19 (32, 47)—or contribute to host defense, as seen in the upregulation of antiviral effector genes like OASL during influenza infection (48).

Our analysis identified a markedly higher number of DMRs than DhMRs between R- and S-ducklings post-infection. Both DMRs and DhMRs were distributed genome-wide but showed clear enrichment on the three largest autosomes and a strong bias toward intronic regions within genebodies. While promoter methylation is widely recognized as a repressive mark, the functional role of genebody methylation is more nuanced and can be associated with both transcriptional activation and repression depending on genomic and cellular context (17, 49–51). Although promoter methylation differences between resistant and susceptible ducklings were also observed, their effect sizes were substantially smaller than those associated with genebody methylation, consistent with the limited dynamic range of promoter methylation under steady-state conditions (52–54). In our system, genebody 5mC levels were negatively correlated with gene expression, a relationship that may be attributable to the high proportion of intronic DMRs, as intron methylation has previously been linked to transcriptional downregulation in vertebrates (55, 56). Notably, we also observed a positive correlation between genebody 5hmC levels and gene expression, suggesting that 5hmC may promote transcription. This finding is consistent with observations in the brain and embryonic stem cells, where 5hmC regulates transcription and cell fate (57–59). However, the effect of 5hmC on transcriptional output is much weaker than that of 5mC, which is likely due to its transient nature (60). In contrast, 5mC exerts a stable and persistent regulatory effect in maintaining DNA methylation patterns.

Functional enrichment of DMR-associated genes in S-ducklings highlighted key pathways potentially governing the duckling’s susceptibility to DHAV-3. A central finding was the link between DNA methylation and the endocytosis pathway. S-ducklings exhibited hypomethylation (and thus likely higher expression) of endocytosis-related genes, whereas R-ducklings showed hypermethylation of the same pathways. This epigenetic configuration suggests that susceptible individuals may possess a more permissive endocytic environment for viral entry. We validated this hypothesis experimentally by showing that pharmacologic inhibition of DNA methylation with 5′AzaC enhanced DHAV-3 uptake in primary hepatocytes. Viral entry is a critical early step in infection (61, 62): while some viruses directly penetrate cell membranes, most rely on endocytosis and vesicular transport (63). These results indicate that methylation-mediated suppression of endocytosis may serve as a protective mechanism in resistant hosts, limiting initial viral entry—a critical bottleneck in the infection cycle.

Beyond entry, DNA methylation was also implicated in regulating downstream host responses. Host cells recognize viruses via pattern recognition receptors and antigen-presenting cells, activating innate and adaptive immunity to clear infections (64–66)—though uncontrolled immune responses can cause severe pathological damage (64, 67). In infected S-ducklings, the enrichment of hypomethylated genes in immune pathways may reflect a heightened antiviral defense but may also underlie the severe immunopathology and acute liver damage observed in these animals. Conversely, hypermethylated genes in S-ducklings were associated with fatty acid metabolism and autophagy—two processes often co-opted by viruses to support replication (68–72). Pharmacologic modulation confirmed that both fatty acid oxidation and autophagy promote DHAV-3 replication. Intriguingly, in our model, these pro-viral pathways were epigenetically repressed (hypermethylated) in S-ducklings, implying that the host may attempt to restrict infection by silencing them. This presents a complex picture in which DNA methylation simultaneously dampens beneficial antiviral defenses (e.g., by limiting endocytosis) while also attempting to inhibit pathways that viruses exploit (e.g., metabolism and autophagy).

An important question raised by our study is whether the pre-existing methylation differences between resistant and susceptible ducklings are genetically encoded or acquired independently of genetic variation. Given that these lines were derived through eight generations of artificial selection (37, 38), it is plausible that underlying genetic variation—potentially acting through methylation quantitative trait loci—contributes to the observed epigenetic divergence (73–75). Alternatively, these signatures could represent stochastic epigenetic events stabilized through development. Future work integrating whole-genome sequencing with methylome analysis across generations will be necessary to resolve this question. Nonetheless, the stability and functional relevance of these pre-existing markers position them as valuable epigenetic biomarkers for resistance breeding, regardless of their genetic or epigenetic origins.

**Fig 11:**
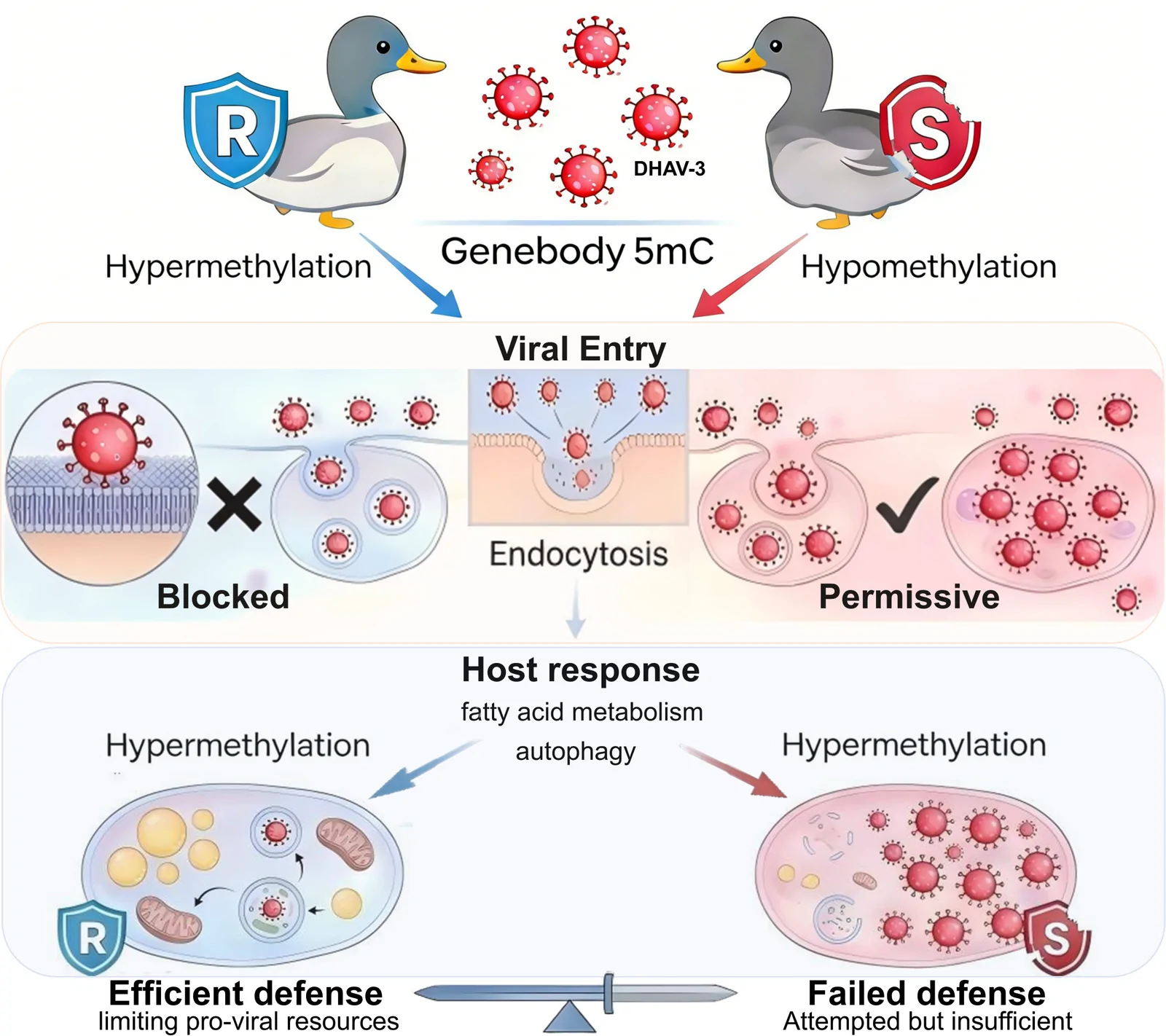
DNA methylation landscapes underlie differential susceptibility to DHAV-3 in ducklings.

In conclusion, our study demonstrates that divergent DNA methylation landscapes underlie differential susceptibility to DHAV-3 in ducklings (Fig. 11). These epigenetic differences shape critical stages of infection, from viral entry via endocytosis to subsequent host metabolic and autophagic responses. The data suggest that host DNA methylation acts as a double-edged sword, with some modifications potentially protective and others contributing to susceptibility. The dual role of DNA methylation becomes evident when examining its pathway- and line-specific effects. In resistant ducklings, hypermethylation of endocytosis-related genes establishes a protective barrier at viral entry, while concurrent hypermethylation of fatty acid metabolism and autophagy pathways further reinforces resistance by limiting pro-viral host resources. In susceptible ducklings, however, hypomethylation of endocytosis genes renders the host inherently permissive to viral entry—a clear pro-viral configuration. Paradoxically, despite this permissiveness, susceptible ducklings maintain hypermethylation of metabolic/autophagy pathways identical to resistant ducklings, suggesting an attempted host defense that is ultimately insufficient. Thus, DNA methylation functions as a context-dependent epigenetic checkpoint whose impact—anti- or pro-viral—is determined by the specific pathways affected and the host genetic background. Further dissection of the key regulatory nodes within this epigenetic network may identify novel targets for intervening in viral pathogenesis, offering strategies to enhance host resistance while mitigating immunopathological damage.

### Conclusion

This study establishes that a pre-configured, intragenic DNA methylation landscape governs innate susceptibility to DHAV-3, primarily by setting the baseline for viral entry via endocytic pathway regulation. Beyond entry, this methylation program coordinates immune, metabolic, and autophagic responses, revealing a multi-layered epigenetic framework that shapes infection outcomes. These findings not only advance our understanding of host–pathogen epigenetic interactions but also provide actionable targets for antiviral intervention and epigenetic marker-assisted breeding in poultry.

## MATERIALS AND METHODS

### Animals and viruses

The DHAV-3-resistant (R) and susceptible (S) ducklings were derived from our previous study (38). The DHAV-3 strain (isolate 112803) was originally isolated from 1-week-old ducklings during a duck viral hepatitis outbreak in China in 2011 and was provided by the Key Laboratory of Animal Epidemiology of the Ministry of Agriculture, China. Seven-day-old ducklings were subcutaneously inoculated with 0.5 mL of DHAV-3 (2.5 × 106.83 ELD50/0.2 mL). Individuals with post-infection survival rates >90% (R) or <30% (S) were selected for further study. For each experimental group, three biologically independent ducklings were used: uninfected resistant (R0, *n* = 3), uninfected susceptible (S0, *n* = 3), infected resistant (R24, *n* = 3), and infected susceptible (S24, *n* = 3). All samples were collected at 0 hpi (pre-infection) and 24 hpi. Liver tissues were collected, snap-frozen in liquid nitrogen, and stored until DNA extraction. Liver samples were also fixed in 4% paraformaldehyde for paraffin sectioning and HE staining for histological examination (Olympus, Japan).

### Whole-genome bisulfite sequencing and oxidative whole-genome bisulfite sequencing

Genomic DNA was extracted using a commercial kit (Genfine, China). DNA quality was assessed using the NanoPhotometer (IMPLEN, USA) and Qubit 2.0 fluorometer (Life Technologies, USA). Libraries were constructed using the Single Strand Bisulfite-Seq Library Prep Kit (Acegen, China) following the manufacturer’s protocol. Briefly, 1 μg of genomic DNA was spiked with 0.1% unmethylated λDNA and sonicated into 200–500 bp fragments. For WGBS, DNA was treated with bisulfite, while oxWGBS samples underwent oxidation prior to bisulfite conversion (TrueMethyl oxBS Module). DNA was thermally denatured to form single strands ligated to the 3′-dA-tailed adapters, followed by extension and ligation of 5′-methylcytosine-modified adapters. Libraries were amplified by ~10 cycles of PCR using 8-bp dual index primers (Illumina, USA) and quality-checked on an Agilent 2100 Bioanalyzer (Agilent Technologies, USA). Sequencing was performed on an Illumina HiSeq X Ten platform with 150-bp paired-end reads (Illumina, USA).

### Data processing

Raw reads were initially quality-assessed using FastQC (v0.12.0) (76). Adapter removal and low-quality data filtering were performed with Trimmomatic (v0.39) (77) to generate clean data with the following parameters: (i) truncated reads when the average base quality of a window <15; (ii) removed reads with head/tail quality <3 or containing Ns; (iii) truncate adapter-contaminated reads; (iv) discarded reads <36 nt post-trimming; (v) discarded the paired failed reads. Clean reads were aligned to the Pekin duck reference genome (GCF_015476345.1) using BSMAP (v2.90) (78). Cytosine methylation levels (β value) were calculated as mC/(mC + umC) using the BSMAP python script methratio.py (mC = methylated cytosines; umC = unmethylated cytosines). Hydroxymethylation sites were identified by subtracting methylation signals between matched WGBS and oxWGBS libraries to construct a hydroxymethylation site matrix for subsequent analysis.

To analyze methylation across functional genomic regions, cytosine sites were extracted from upstream 2k, 5′UTR, exons, introns, 3′UTR, and downstream 2k regions. Each region was divided into 20 bins, and the average β value was computed per bin. DMR and DhMR were identified using Metilene (v0.2.8) (79) with the following thresholds: inter-CpG distance < 300 nt; number of CpGs > 5; |Δβ| > 0.1 (other parameters default).

### Integration with transcriptome data

To examine the relationship between DNA methylation/hydroxymethylation and gene expression, DMRs and DhMRs located in promoter or genebody regions were correlated with transcript levels from matched full-length transcriptome data. Given the limited number of genes anchored by DMRs/DhMRs under strict thresholds (especially in R0 and S0), we applied a relaxed cutoff (|Δβ| > 0) to capture more biologically relevant information. Δβ values were plotted against log2(fold change) in gene expression using the ggplot2 package in R (80), and associations were assessed via Spearman’s correlation analysis. Groups were defined based on the sign of Δβ: 5mC-high/5hmC-high (Δβ > 0 in DMR/DhMR); 5mC-low/5hmC-low (Δβ < 0 in DMR/DhMR). Similarly, the anchoring regions were also grouped: promoter-high/genebody-high (Δβ > 0 in promoter/genebody) and promoter-low/genebody-low (Δβ < 0 in promoter/genebody). Gene expression changes were presented as log₂(fold change) values. Statistical analyses were performed using the Wilcoxon rank-sum test and Kruskal–Wallis test, with post hoc Dunn’s test for multiple comparisons.

### Functional enrichment analysis

Genebody DMR-anchored genes were selected for enrichment analysis to identify biological pathways associated with DNA methylation alterations. Gene Ontology (GO) and Kyoto Encyclopedia of Genes and Genomes (KEGG) annotations were performed using the Database for Annotation, Visualization, and Integrated Discovery (81). Duck homologous gene information was extracted using the org.Gg.eg.db package (82). ssGSEA of the endocytosis pathway gene set (gga04144) was conducted with the GSEABase and GSVA packages (83, 84). Statistical tests were performed using the Wilcoxon rank-sum test.

### Cell culture

Primary duck hepatocytes were isolated from 14-day-old Pekin duck embryos. Briefly, embryos were disinfected, and livers were isolated and washed in ice-cold phosphate-buffered saline (PBS) containing 1% sodium heparin (Biosharp, China). Liver tissues were minced after removal of fibrous membranes and gallbladders, and washed three times with PBS containing 1% bovine serum albumin (Solarbio, China). Tissue fragments were digested with 2 mg/mL type II collagenase (Sigma-Aldrich, Germany) at 37°C for 20 min. Digestion was terminated with 3 volumes of DMEM (Gibco, USA) supplemented with 10% fetal bovine serum (BioChannel, China). Cell suspensions were filtered through 100 and 70 μm cell strainers (Biosharp, China), centrifuged at 900 × *g* for 5 min, and erythrocytes were lysed using lysis buffer (Solarbio, China). Cells were seeded at a density of 2.5 × 10^5^ cells/mL in high-glucose DMEM containing 10% FBS, 100 U/mL penicillin, and 0.1 mg/mL streptomycin, and maintained at 37°C under 5% CO₂.

### Drug treatment and viral infection

Cytotoxicity of 5-Azacytidine, Etomoxir, Rapamycin, and Chloroquine (MCE, China) was determined using a CCK-8 assay (Beyotime, China). Cells were pretreated with non-cytotoxic drug concentrations for 6 h, followed by inoculation with DHAV-3 (10⁴ copies/μL, 100 μL per well). Drugs were halved with virus infection to maintain interference. After adsorption at 4°C for 30 min, the unbound virus was removed by washing with cold PBS. Cultures were maintained in DMEM with 2% chicken serum (Solarbio, China). Samples were collected at 1.5 hpi for 5-Azacytidine treated cells and 24 hpi for other drug-treated groups.

### Transmission electron microscopy

Cells were fixed in 2.5% glutaraldehyde for 5 min at room temperature (Biosharp, China), scraped, and centrifuged at 3000 × *g* for 2 min. Pellets were refixed in fresh glutaraldehyde for 30 min in the dark, followed by overnight fixation at 4°C. Samples were washed three times with 0.1 M PBS (15 min each), post-fixed in 1% OsO₄ for 2 h, and washed again. Gradient dehydration was performed with 50%, 70%, 80%, 90%, 95%, 100% ethanol (15 min each, 100% ethanol repeated), followed by 100% acetone (15 min each, repeated). Samples were infiltrated with acetone (SCRC, China):EMBed 812 (SPI, USA) mixtures (1:1 for 4 h, 2:1 for 8 h) and pure EMBed 812 (overnight), then polymerized at 60°C for 48 h. Ultrathin sections (60–80 nm) were cut by ultramicrotome (Leica UC7, Germany), stained with uranyl acetate and citrate (15 min each), rinsed with distilled water, and air-dried overnight. Sections were imaged on a transmission electron microscope (TEM) (Hitachi HT7700, Japan). Panels contain magnified regions derived from the same original TEM field per treatment condition (DMSO control and 5′AzaC-treated, respectively), selected to illustrate viral entry events. Endocytosed viral particles were quantified using ImageJ (v1.49).

### Real-time PCR

Total RNA was extracted from cells and tissues (liver, spleen, kidneys, and bursa of Fabricius) using RNAiso Plus (Takara, Japan). cDNA was synthesized with HiScript IV SuperMix (Vazyme, China), and qPCR was performed using ChamQ BlueSYBR qPCR Master Mix (Vazyme, China). Viral load and expression levels of *IFN-γ*, *TNF-α*, *IL-6*, *IL-17A*, *ACADM*, *CPT1A*, *PPARα*, *MAP1LC3B*, *LAMP1,* and *ATG16L* were calculated using the 2^−ΔΔct^ method. Statistical analyses were performed using the Wilcoxon rank-sum test. All primers are listed in Table S1.

## Supplementary Material

Reviewer comments

## Data Availability

Raw fastq files generated in this study have been deposited in the China National Center for Bioinformation (https://www.cncb.ac.cn/) with the project number PRJCA056211.

## SUPPLEMENTAL MATERIAL

Fig. S1 spectrum.00565-26-s0001.pdf The distribution of the lengths of DMRs and DhMRs between R- and S-ducklings.

Fig. S2 spectrum.00565-26-s0002.pdf Integration analysis of DNA methylation and hydroxymethylation with gene expression between uninfected and infected ducklings.

Fig. S3 spectrum.00565-26-s0003.pdf Functional enrichment analysis of DMGs in genebody between uninfected and infected ducklings.

Supplemental material spectrum.00565-26-s0004.docx Supplemental figure and table legends.

Supplemental tables spectrum.00565-26-s0005.xlsx Tables S1 to S6.

OPEN PEER REVIEW reviewer-comments.pdf An accounting of the reviewer comments and feedback.

ASM does not own the copyrights to Supplemental Material that may be linked to, or accessed through, an article. The authors have granted ASM a non-exclusive, world-wide license to publish the Supplemental Material files. Please contact the corresponding author directly for reuse.
