# Supplementary figures and images for: DNA methylation-mediated suppression of endocytosis confers resistance to duck hepatitis A virus type 3
Source: Microbiol Spectr. 2026 Jun 15;14(7):e00565-26. doi: 10.1128/spectrum.00565-26 (PMC13340123; doi:10.1128/spectrum.00565-26)

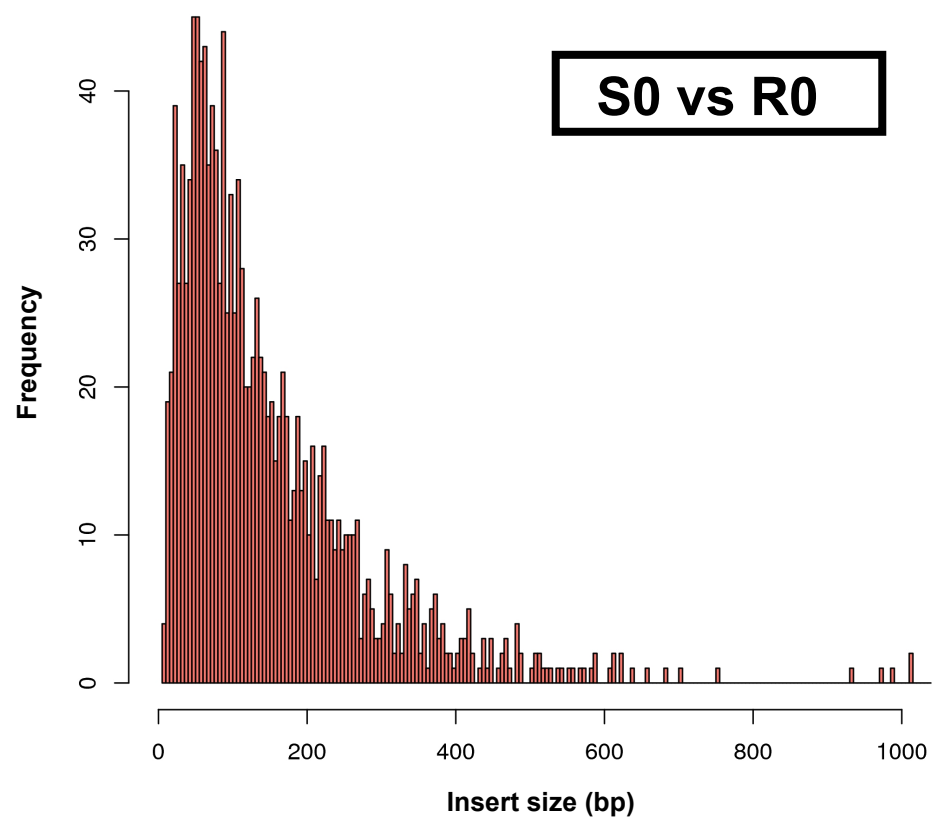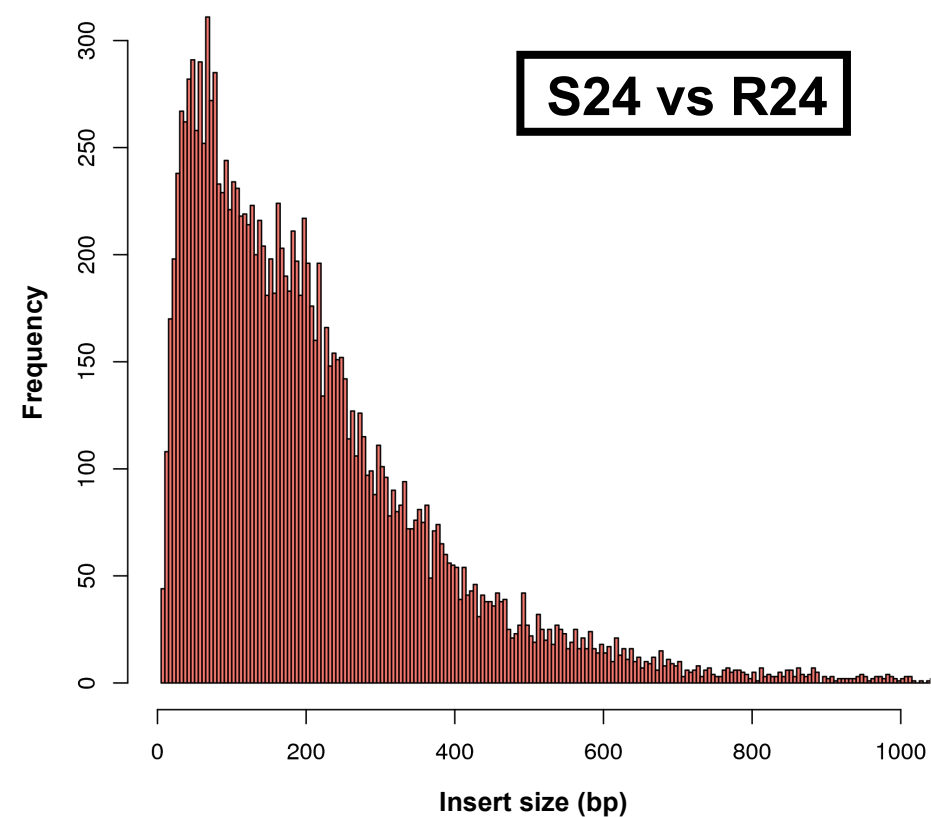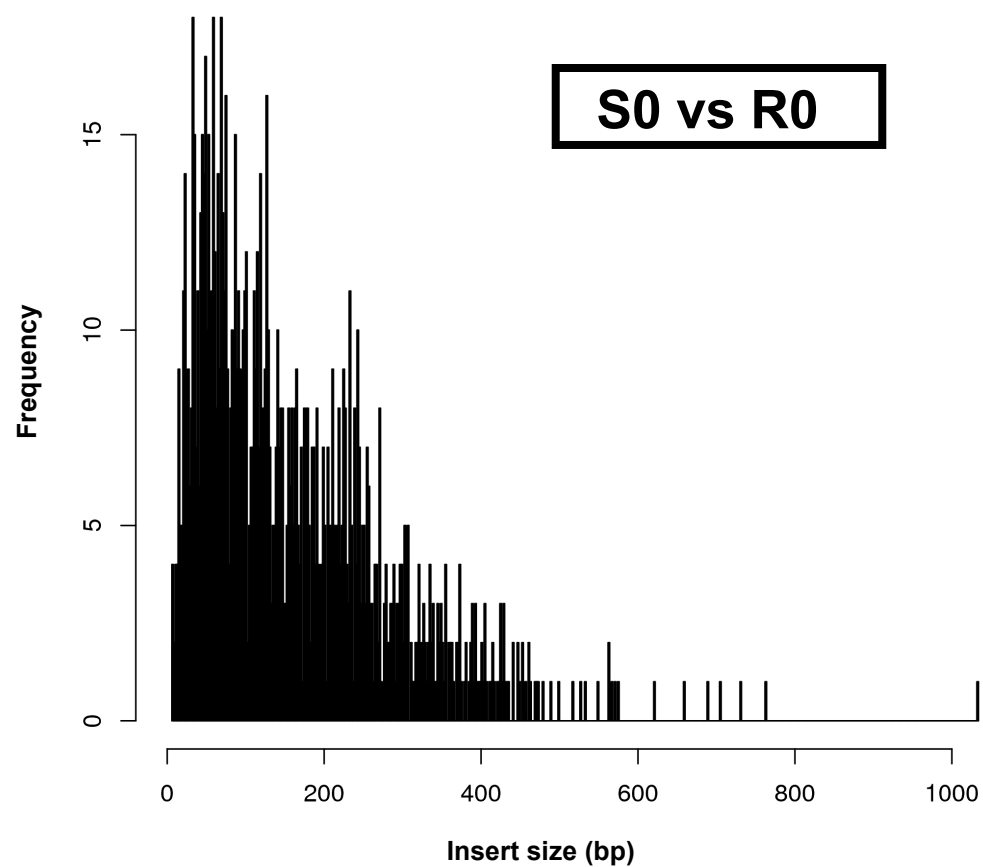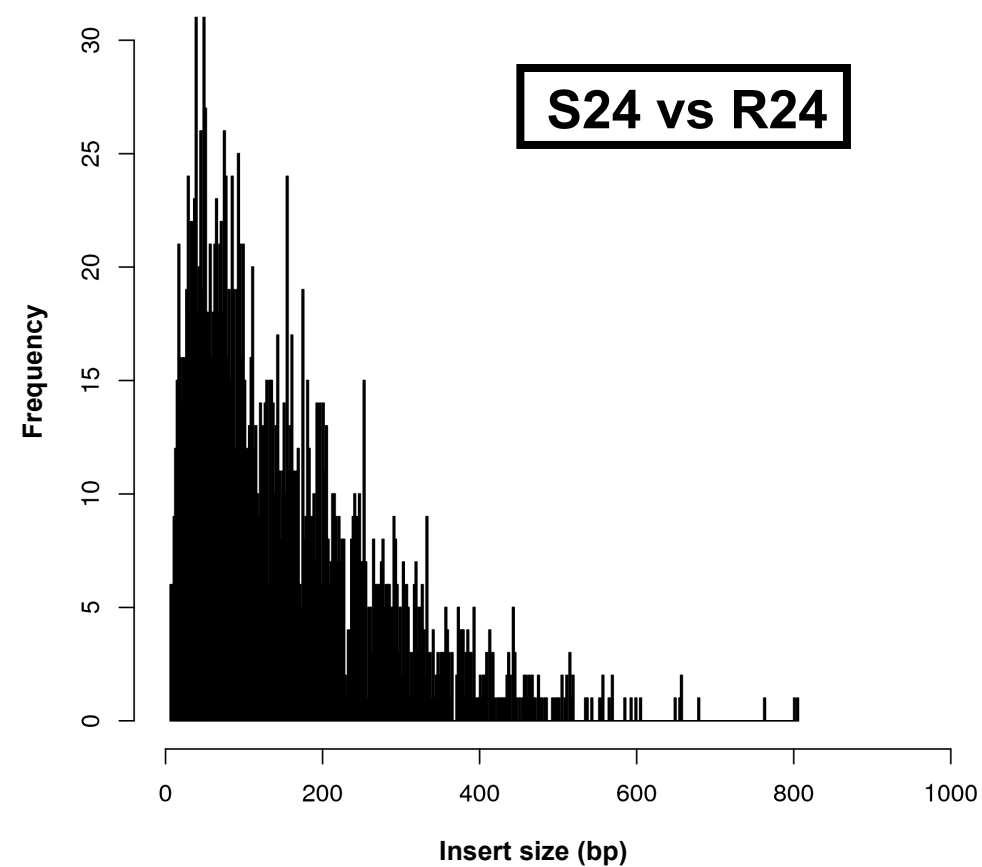

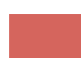 **DMR**

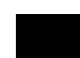 **DhMR**

Supplement: Fig. S1 — The distribution of the lengths of DMRs and DhMRs between R- and S-ducklings. [file spectrum.00565-26-s0001.pdf]

**R24 vs R0**

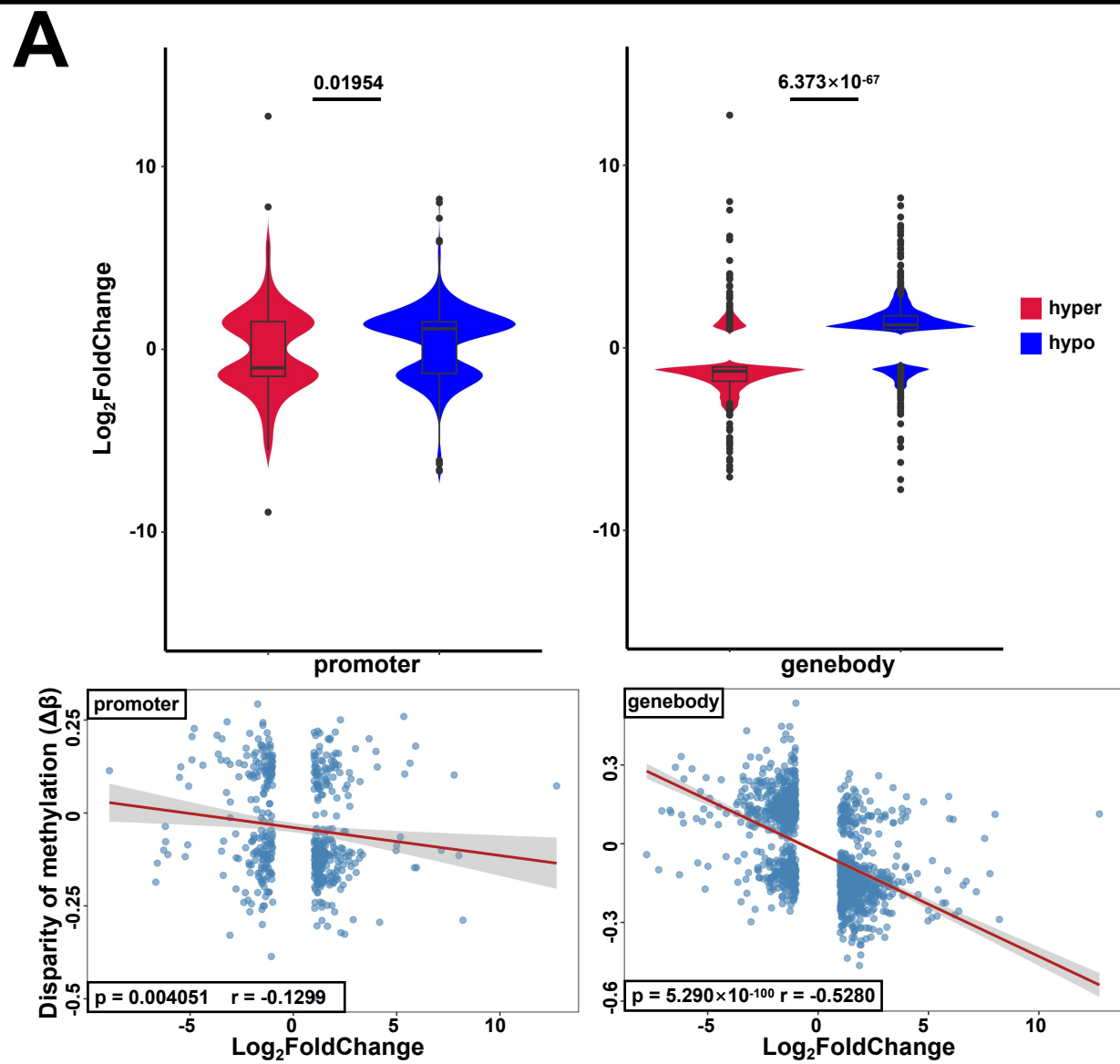

**S24 vs S0**

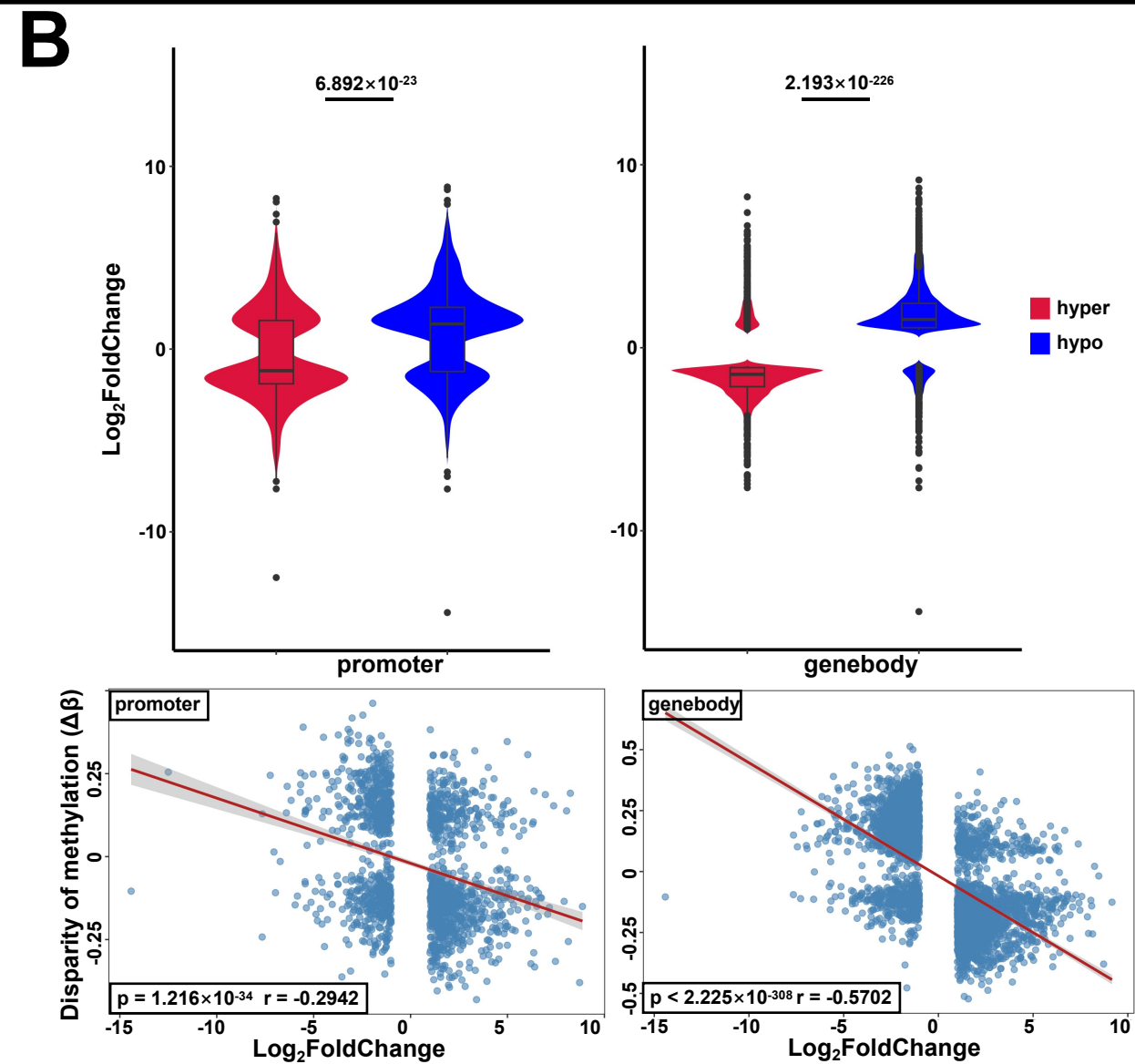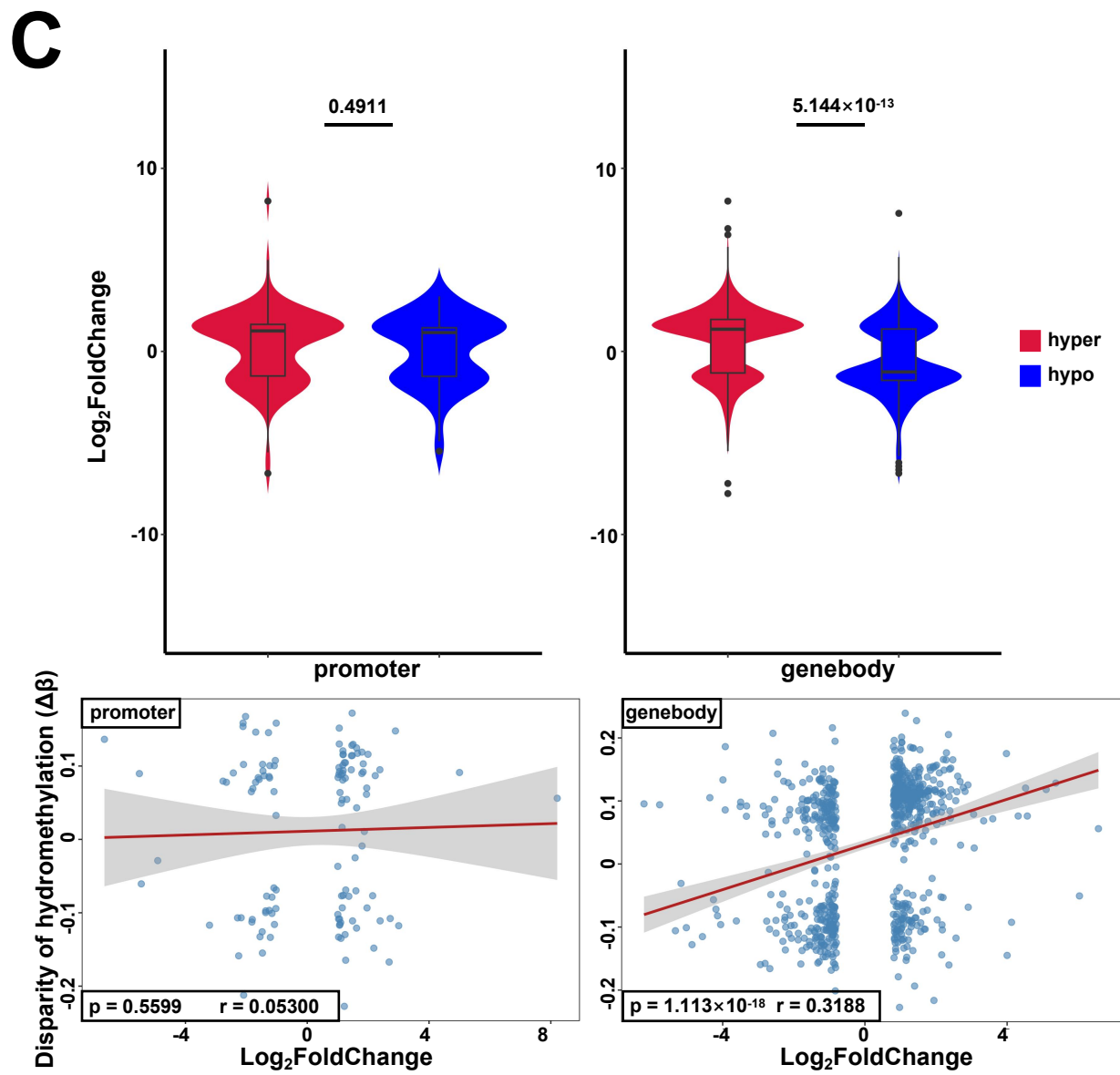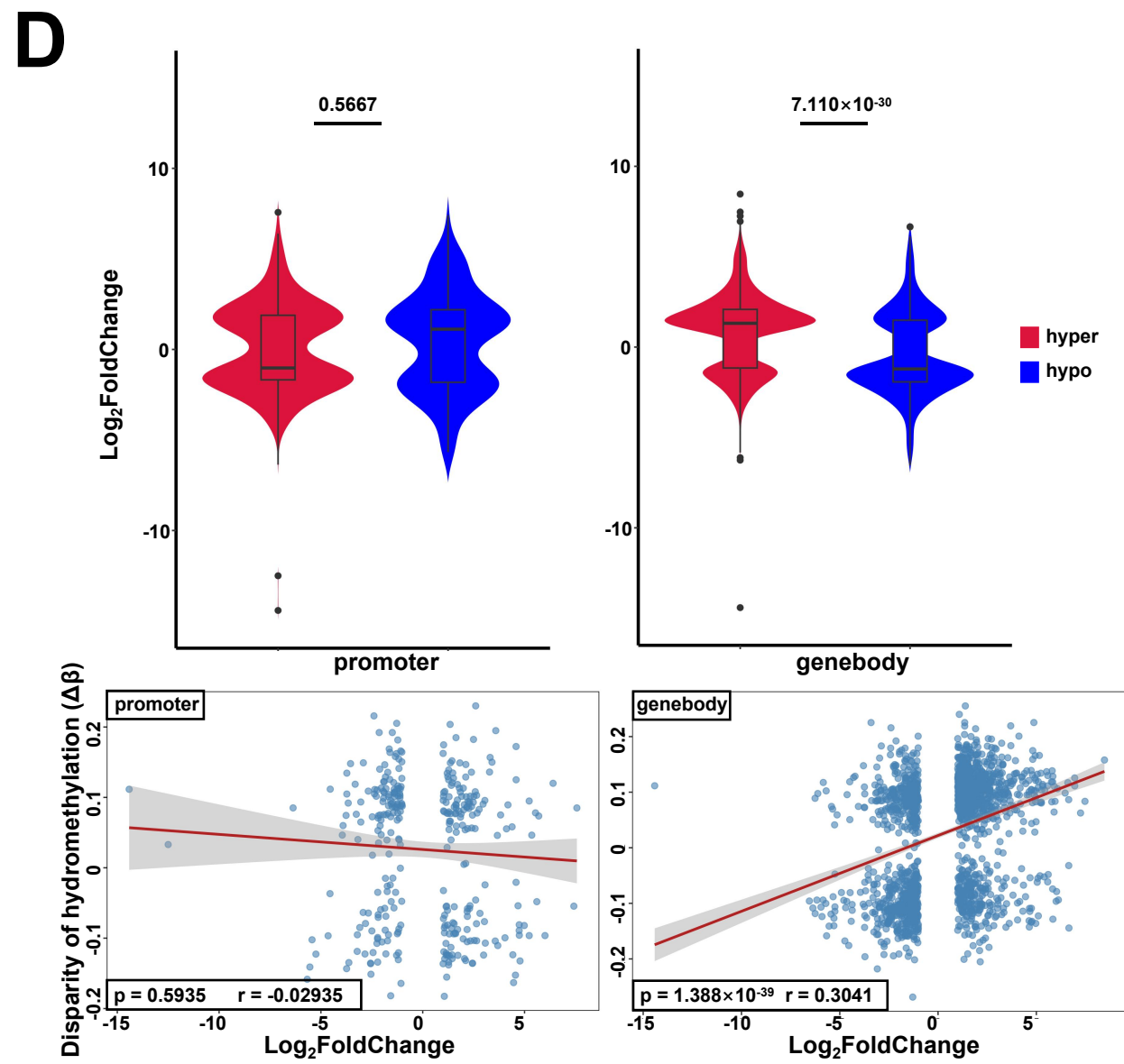

Supplement: Fig. S2 — Integration analysis of DNA methylation and hydroxymethylation with gene expression between uninfected and infected ducklings. [file spectrum.00565-26-s0002.pdf]

R24 vs R0

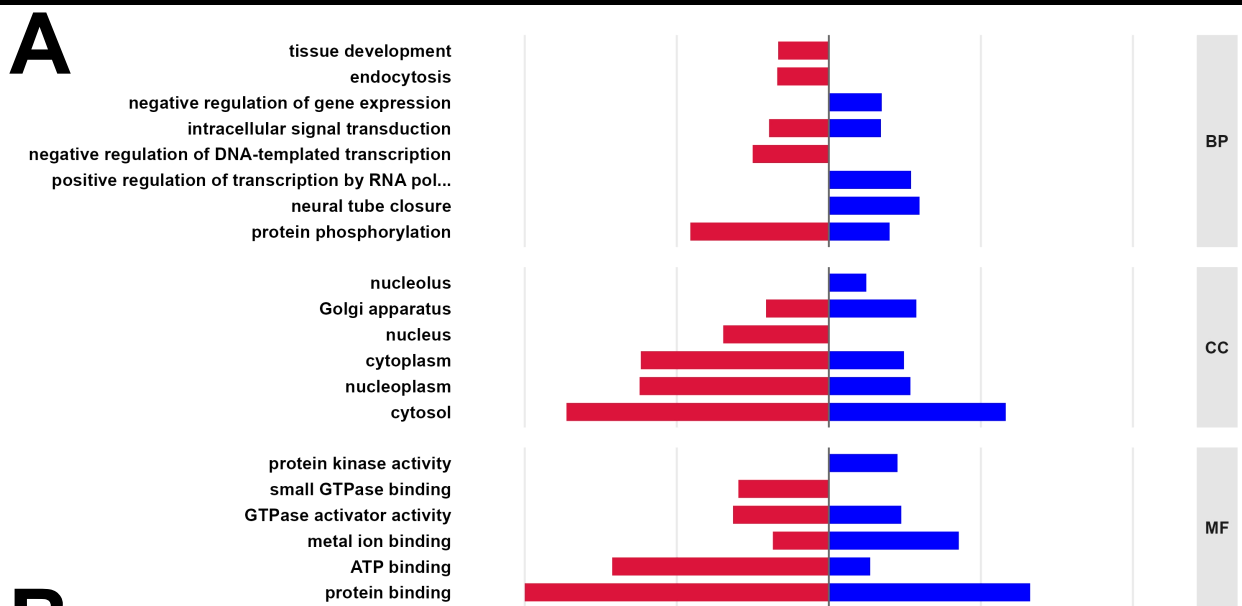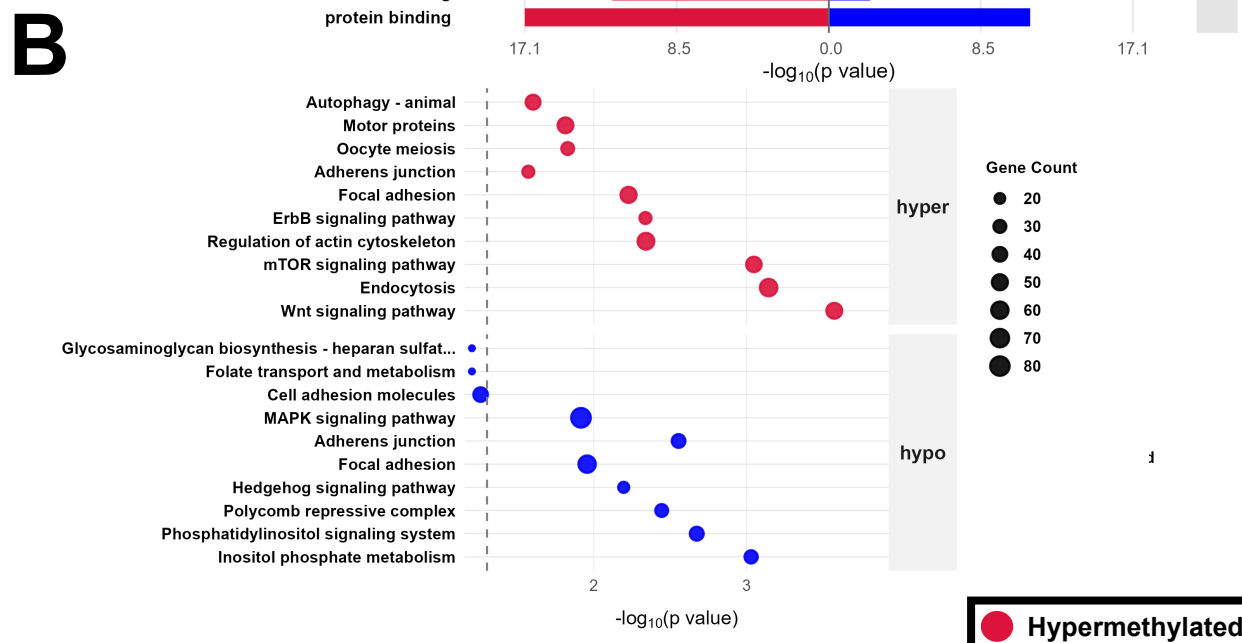

S24 vs S0

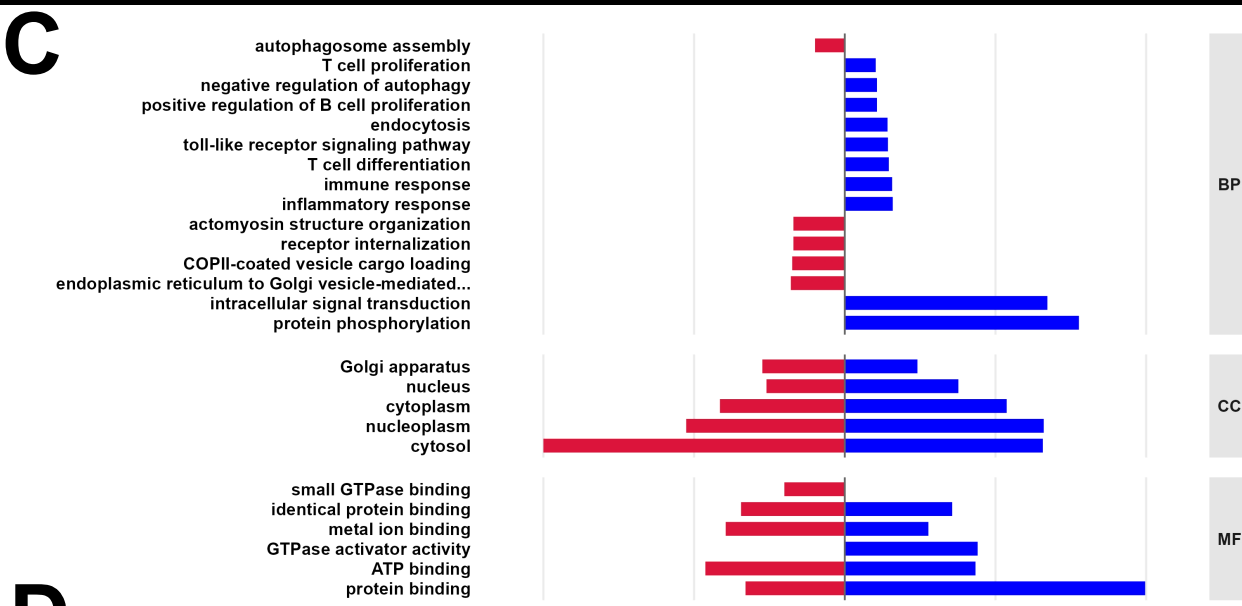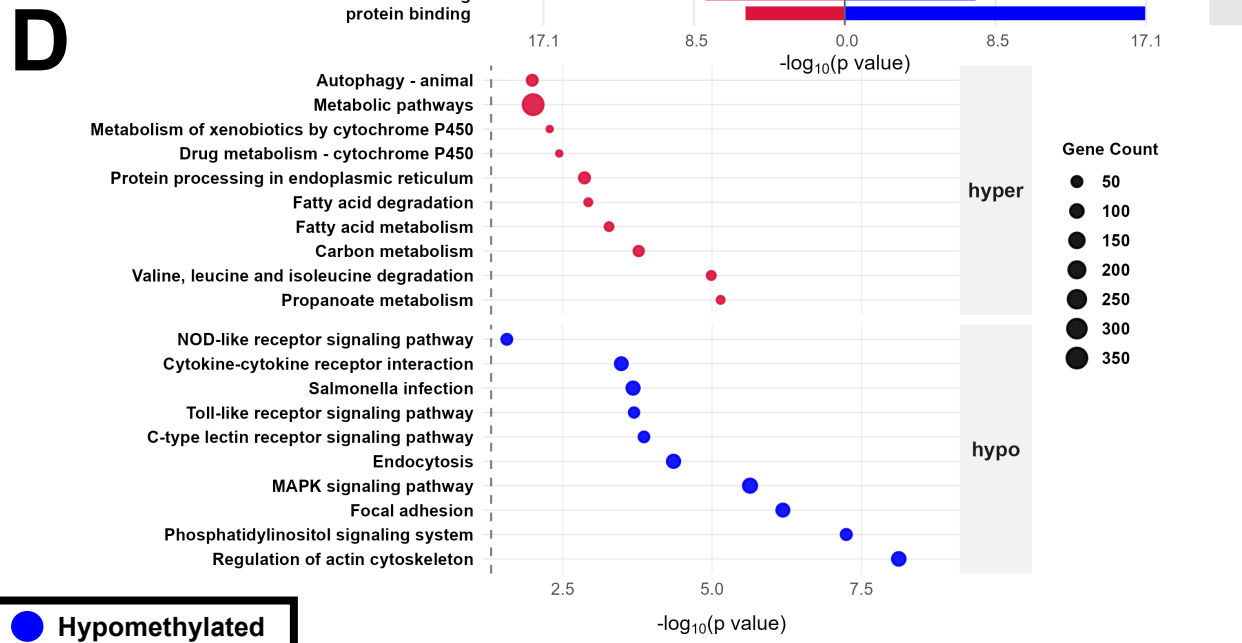

Supplement: Fig. S3 — Functional enrichment analysis of DMGs in genebody between uninfected and infected ducklings. [file spectrum.00565-26-s0003.pdf]
