# Supplementary material for: DNA methylation-mediated suppression of endocytosis confers resistance to duck hepatitis A virus type 3
Source: Microbiol Spectr. 2026 Jun 15;14(7):e00565-26. doi: 10.1128/spectrum.00565-26 (PMC13340123; doi:10.1128/spectrum.00565-26)
Supplement: Supplemental material — Supplemental figure and table legends. [file spectrum.00565-26-s0004.docx]

**Figure legends**

**Figure S1 The distribution of the lengths of DMRs and DhMRs between R- and S-ducklings.**

**Figure S2 Integration analysis of DNA methylation and hydroxymethylation with gene expression between uninfected and infected ducklings.**

The correlation between mRNA expression changes and methylation levels of DMGs in R-ducklings (**a**) and S-ducklings (**b**) during DHAV3 infection. The correlation between mRNA expression changes and methylation levels of DhMGs in R-ducklings (**c**) and S-ducklings (**d**).

**Figure S3 Functional enrichment analysis of DMGs in genebody between uninfected and infected ducklings.**

GO enrichment analysis of hyper- and hypo-DMGs in R-ducklings (**a**) and S-ducklings (**c**) during DHAV3 infection. Functional clusters of KEGG for hyper- and hypo-DMGs in R-ducklings (**b**) and S-ducklings (**d**)

**Table S1 Primer sequences for qPCR.**

**Table S2 Summary of different cytosine methylation-rate in oxWGBS and WGBS libraries.**

**Table S3 The information of differentially methylated regions (DMRs) in CG contexts.**

(**a**) DMRs between R0 and S0 samples. (**b**) DMRs between R24 and S24 samples.

**Table S4 The information of differentially hydroxymethylated regions (DhMRs) in CG contexts.**

(**a**) DhMRs between R0 and S0 samples. (**b**) DhMRs between R24 and S24 samples.

**Table S5 Gene Ontology (GO) enrichment of DMRs in promoter and genebody identified through integrative analysis.**

**Table S6 Kyoto Encyclopedia of Genes and Genomes (KEGG) enrichment of DMRs in promoter and genebody identified through integrative analysis.**
