# Supplementary material for: DNA methylation-mediated suppression of endocytosis confers resistance to duck hepatitis A virus type 3
Source: Microbiol Spectr. 2026 Jun 15;14(7):e00565-26. doi: 10.1128/spectrum.00565-26 (PMC13340123; doi:10.1128/spectrum.00565-26)
Supplement: Reviewer comments [file reviewer-comments.pdf]

AMERICAN SOCIETY FOR MICROBIOLOGY | Microbiology Spectrum

DNA Methylation-Mediated Suppression of Endocytosis Confers Resistance to Duck Hepatitis A Virus Type 3

Shaofei Li, Di Hu, Xiang Mei, Lionel Kinkpe, Shuaiqin Wang, Jie Wei, Shuisheng Hou, Yunsheng Zhang, and Xia Wang

Corresponding Author(s): Xia Wang, Northwest A&F University

Review Timeline:

Submission Date:	February 15, 2026
Editorial Decision:	March 23, 2026
Revision Received:	March 28, 2026
Accepted:	April 9, 2026

Editor: Zsolt Toth

Reviewer(s): The reviewers have opted to remain anonymous.

Transaction Report:

(Note: With the exception of the correction of typographical or spelling errors that could be a source of ambiguity, letters and reports are not edited. The original formatting of letters and referee reports may not be reflected in this compilation.)

DOI: https://doi.org/10.1128/spectrum.00565-26

Re: Spectrum00565-26 (**DNA Methylation-Mediated Suppression of Endocytosis Confers Resistance to Duck Hepatitis A Virus Type 3**)

Dear Prof. Xia Wang:

Thank you for the privilege of reviewing your work. Below you will find my comments, instructions from the Spectrum editorial office, and the reviewer comments. The reviewers recognized the study's importance but noted areas of the manuscript that require improvement. Please revise the manuscript in accordance with the review recommendations. Your revisions should address the specific points made by each reviewer.

Please return the manuscript within 60 days; if you cannot complete the modification within this time period, please contact me. If you do not wish to modify the manuscript and prefer to submit it to another journal, notify me immediately so that the manuscript may be formally withdrawn from consideration by Spectrum.

Revision Guidelines
To submit your modified manuscript, log into the submission site at https://spectrum.msubmit.net/cgi-bin/main.plex. Go to Author Tasks and click the appropriate manuscript title to begin. The information you entered when you first submitted the paper will be displayed; update this as necessary. Note the following requirements:

• Upload point-by-point responses to the issues raised by the reviewers in a file named "Response to Reviewers," NOT in your cover letter.
• Upload a compare copy of the manuscript (without figures) as a "Marked-Up Manuscript" file.
• Upload a clean .DOC/.DOCX version of the revised manuscript and remove the previous version.
• Each figure must be uploaded as a separate, editable, high-resolution file (TIFF or EPS preferred), and any multipanel figures must be assembled into one file.
• Any supplemental material intended for posting by ASM should be uploaded with their legends separate from the main manuscript. You can combine all supplemental material into one file (preferred) or split it into a maximum of 10 files with all associated legends included.

For complete guidelines on revision requirements, see our Submission and Review Process webpage. Submission of a paper that does not conform to guidelines may delay acceptance of your manuscript.

Data availability: ASM policy requires that data be available to the public upon online posting of the article, so please verify all links to sequence records, if present, and make sure that each number retrieves the full record of the data. If a new accession number is not linked or a link is broken, provide Spectrum production staff with the correct URL for the record. If the accession numbers for new data are not publicly accessible before the expected online posting of the article, publication may be delayed; please contact production staff (Spectrum@asmusa.org) immediately with the expected release date.

Publication Fees: For information on publication fees and which article types are subject to charges, visit our website. If your manuscript is accepted for publication and any fees apply, you will be contacted separately about payment during the production process; please follow the instructions in that e-mail. Arrangements for payment must be made before your article is published.

ASM Membership: Corresponding authors may join or renew ASM membership to obtain discounts on publication fees. Need to upgrade your membership level? Please contact Customer Service at Service@asmusa.org.

The ASM Journals program strives for constant improvement in our submission and publication process. Please tell us how we can improve your experience by taking this quick Author Survey.

Thank you for submitting your paper to Spectrum.

Sincerely,
Zsolt Toth
Editor
Microbiology Spectrum

Reviewer #1 (Comments for the Author):

Comments and Suggestions for Authors
This manuscript presents a comprehensive and well-executed study investigating the role of DNA methylation in determining susceptibility to duck hepatitis A virus type 3 (DHAV-3). The integration of whole-genome bisulfite sequencing (WGBS),

oxidative bisulfite sequencing (oxWGBS), and transcriptomics, combined with functional validation using pharmacological inhibitors, provides a robust dataset. The identification of a pre-existing intragenic DNA methylation signature that regulates viral entry via the endocytic pathway is a novel and significant finding. However, several points could be addressed to further strengthen the manuscript prior to publication.

Major Comments:

1. Clarity on the "pre-existing" epigenetic signature:

The authors convincingly demonstrate that methylation differences between resistant and susceptible ducklings exist prior to infection. However, the origin of these differences-whether they are genetically determined or acquired-remains unclear. A brief discussion on the potential heritability of this epigenetic signature or its relationship to underlying genetic variation (e.g., methylation quantitative trait loci, meQTLs) would add depth and contextualize the findings within the broader framework of epigenetic inheritance and selective breeding.

2. Clarity on 5mC vs. 5hmC contributions:

The distinction between 5mC and 5hmC is a strength of the study. However, the conclusion that 5mC, not 5hmC, drives expression changes (Fig. 6) is based on grouped analyses. It would be helpful to include a more detailed discussion of why 5hmC may not correlate with expression in this context, especially given its known role in gene regulation in other systems. Could this be tissue-specific or related to the timing of infection?

Minor Comments:

1. Keywords optimization: Line 49. The current keywords ("DNA methylation; Viral susceptibility; Endocytosis; virus; DHAV-3") are appropriate but could be more specific for indexing purposes. Consider replacing "virus" with "Host-pathogen interaction" or "Epigenetics," and adding "Duck" or "Avian" to increase discoverability for readers interested in poultry or animal models.

2. Results section organization: In section 2.4, the authors describe the correlation between 5mC/5hmC and gene expression, followed by subgroup analyses in section 2.5. The transition between these sections is smooth, but the logic could be further clarified by adding a brief introductory sentence at the beginning of section 2.5, such as: "Having established that genebody 5mC is the dominant regulatory layer, we next investigated the biological pathways associated with these methylation changes." This would help guide readers through the narrative.

3. Terminology consistency: Line 196-202. In the Results (section 2.4), the terms "genebody-high" and "genebody-low" are used, while in the Discussion, "intragenic methylation" is introduced. To avoid confusion, consider defining "intragenic methylation" as methylation within genebodies (including introns and exons) and using this term consistently throughout the manuscript, or at least clarifying the relationship between these terms when first introduced.

4. Figure 6 legend clarity: Line 204-211. In Fig. 6a and 6b, the four groups are labeled as "5mC-high/5hmC-high," "5mC-high/5hmC-low," etc. This is clear but lengthy. Consider defining these as "H/H," "H/L," "L/H," "L/L" in the figure with an explanatory footnote in the legend (e.g., "H: hypermethylated/hydroxymethylated; L: hypomethylated/hydroxymethylated") to reduce visual clutter.

6. Gene name formatting: Line 270-271. In the text and figures, gene names (e.g., ACADM, CPT1A, PPARα) are correctly italicized. However, in some figure legends (e.g., Fig. 10b, 10c), gene names are not italicized. Please ensure consistent formatting throughout, following journal guidelines (typically italics for genes).

7. Discussion length: The Discussion is comprehensive but somewhat lengthy. Consider condensing the first paragraph, which broadly introduces virus-host interactions and DNA methylation, as much of this context is already covered in the Introduction. This would create more space to expand on the "double-edged sword" concept or the pre-existing signature origin as suggested in Major Comments.

8. Conclusion section redundancy: The Conclusion largely restates key findings from the Discussion. While a summary is valuable, some sentences are nearly identical to those in the Discussion (e.g., "intragenic 5mC modifications, rather than promoter methylation, play a dominant role"). Consider shortening the Conclusion to 3-4 concise sentences that highlight the most novel take-home messages without repeating detailed results.

9. Methods section 5.6 cell culture medium composition: Line 461. The cell culture medium is described as "DMEM containing 10% FBS, 100 U/mL penicillin, and 0.1 mg/mL streptomycin." It would be helpful to specify whether this is high-glucose DMEM or another formulation, as hepatocyte viability can be affected by glucose concentration.

10. Methods section 5.7 drug treatment duration: Line 467. The text states that cells were treated with drugs for 6 h prior to infection, then infected and cultured for 1.5 h (5'AzaC) or 24 h (other drugs). It is unclear whether the drugs were present during the infection and post-infection culture period. Please clarify whether drugs were removed after pretreatment or maintained throughout the experiment.

Summary: This is a solid and interesting study that makes a significant contribution to the field of viral epigenetics. The recommended revisions are primarily aimed at enhancing clarity, mechanistic insight, and readability.

Reviewer #2 (Comments for the Author):

The manuscript "DNA Methylation-Mediated Suppression of Endocytosis Confers Resistance to Duck Hepatitis A Virus Type 3" by Li et al., describes an intriguing experimental study that in Duck Hepatitis A Virus Type 3 (DHAV-3) resistant ducklings, increased DNA methylation (5mC) may act as a barrier that limits viral entry via endocytosis, whereas in susceptible duckling, lower methylation levels could make it easier for the virus to infect cells. Furthermore, the authors also demonstrated that DHAV-3 resistant ducklings also exhibit suppression of fatty acid metabolism and authophagy via 5mC, further linking epigenetic regulation to both immune responses and metabolic processes critical during DHAV-3 infection. Overall, these results provide

valuable insights into how epigenetic modifications govern antiviral defenses and underscore the complex interplay between host and pathogen evolution.
The methods used in the manuscript were suitable for the purpose intended. Overall, this is a beautifully crafted manuscript incorporating the validated results and thus offer a substantial contribution to the virology field. Following are the minor comments that would likely improve the quality and readability of the manuscript to the readers:
Lines 112-115: Fig. 1. The legends are not descriptive. Please provide the details of each panel of this figure including the statistical analyses, so that it becomes easier for reader to follow.
Line 118: 'DHAV-3 susceptibility' can be replaced with 'duckling's susceptibility to DHAV-3'. Besides, check this in Lines 215 and 324.
Lines 169-170: In Figure 4(e), it is difficult to differentiate between R0 vs S0 and R24 vs S24. Please use the appropriate symbols that offer better differentiation.
Lines 175-179: It is always preferred to provide the correlation coefficient and significant changes associated p-values within the manuscript text, as the readers will many a times find it difficult to follow these statistical analyses (by looking at the figure separately).
Line 181: Please check the p-value of Figure 5(c) genebody as it is extremely small.
Lines 184-185: It is interesting to observe significant differences between the expression levels of controls (S0 vs R0) in the promoter region (although it is weaker). Have you investigated it?
Line 215: virus susceptibility or host susceptibility? Please check.
Lines 267-274: In Figure 10 (a, g, h, and i), legends on y-axis are not clear. Relative DHAV3 mRNA or Relative DHAV3 viral RNA copies? Please check. 'Con' is non-standard abbreviation of 'control'. It is better to write 'control'. In Figure 10 (b and c), please correct 'leve' on y-axis.
Line 362: I think Figure 11 can be re-structured to truly depict what is happening in your study. For instance, fatty acid metabolism and autophagy (hypermethylated in S-duckling) needs to be emphasized to differentiate between S- and R-ducklings.
Line 383: Please mention exactly from where the virus was procured. China or some other country?
Lines 386-387: Please inform the biological replicates taken by the authors to derive the conclusions as well experimental plan in the methods section to make it clearer to the readers.
Minor topographical corrections- Line 52, 265, 404,

The manuscript “DNA Methylation-Mediated Suppression of Endocytosis Confers Resistance to Duck Hepatitis A Virus Type 3” by Li et al., describes an intriguing experimental study that in Duck Hepatitis A Virus Type 3 (DHAV-3) resistant ducklings, increased DNA methylation (5mC) may act as a barrier that limits viral entry via endocytosis, whereas in susceptible duckling, lower methylation levels could make it easier for the virus to infect cells. Furthermore, the authors also demonstrated that DHAV-3 resistant ducklings also exhibit suppression of fatty acid metabolism and authophagy via 5mC, further linking epigenetic regulation to both immune responses and metabolic processes critical during DHAV-3 infection. Overall, these results provide valuable insights into how epigenetic modifications govern antiviral defenses and underscore the complex interplay between host and pathogen evolution.

The methods used in the manuscript were suitable for the purpose intended. Overall, this is a beautifully crafted manuscript incorporating the validated results and thus offer a substantial contribution to the virology field. Following are the minor comments that would likely improve the quality and readability of the manuscript to the readers:

Lines 112-115: Fig. 1. The legends are not descriptive. Please provide the details of each panel of this figure including the statistical analyses, so that it becomes easier for reader to follow.

Line 118: ‘DHAV-3 susceptibility’ can be replaced with ‘duckling’s susceptibility to DHAV-3’. Besides, check this in Lines 215 and 324.

Lines 169-170: In Figure 4(e), it is difficult to differentiate between R0 vs S0 and R24 vs S24. Please use the appropriate symbols that offer better differentiation.

Lines 175-179: It is always preferred to provide the correlation coefficient and significant changes associated p-values within the manuscript text, as the readers will many a times find it difficult to follow these statistical analyses (by looking at the figure separately).

Line 181: Please check the p-value of Figure 5(c) genebody as it is extremely small.

Lines 184-185: It is interesting to observe significant differences between the expression levels of controls (S0 vs R0) in the promoter region (although it is weaker). Have you investigated it?

Line 215: virus susceptibility or host susceptibility? Please check.

Lines 267-274: In Figure 10 (a, g, h, and i), legends on y-axis are not clear. Relative DHAV3 mRNA or Relative DHAV3 viral RNA copies? Please check. ‘Con’ is non-standard abbreviation of ‘control’. It is better to write ‘control’. In Figure 10 (b and c), please correct ‘leve’ on y-axis.

Line 362: I think Figure 11 can be re-structured to truly depict what is happening in your study. For instance, fatty acid metabolism and autophagy (hypermethylated in S-duckling) needs to be emphasized to differentiate between S- and R-ducklings.

Line 383: Please mention exactly from where the virus was procured. China or some other country?

Lines 386-387: Please inform the biological replicates taken by the authors to derive the conclusions as well experimental plan in the methods section to make it clearer to the readers.

Minor topographical corrections- Line 52, 265, 404,

西北农林科技大学
NORTHWEST A&F UNIVERSITY

Xia Wang
Associate Professor
xiawang@nwafu.edu.cn

College of Animal Science and Technology,
Northwest A&F University,
Yangling, Shaanxi 712100, China

Response to editor and reviewers:

Thank you very much for giving us the opportunity to revise our manuscript. We appreciate the editor and reviewers very much for their positive and constructive comments and suggestions on our manuscript.

*We have studied the comments and suggestions carefully and have made revisions which marked red in the main manuscript (**Marked-Up Manuscript**). The detailed revisions and point-to-point responses to the reviewer's comments are as follows:*

Reviewer 1

1. Clarity on the "pre-existing" epigenetic signature: The authors convincingly demonstrate that methylation differences between resistant and susceptible ducklings exist prior to infection. However, the origin of these differences-whether they are genetically determined or acquired-remains unclear. A brief discussion on the potential heritability of this epigenetic signature or its relationship to underlying genetic variation (e.g., methylation quantitative trait loci, meQTLs) would add depth and contextualize the findings within the broader framework of epigenetic inheritance and selective breeding.

Response: We thank the reviewer for this insightful comment. We have added a new paragraph in the discussion (Line 292-302).

"An important question raised by our study is whether the pre-existing methylation differences between resistant and susceptible ducklings are genetically encoded or acquired independently of genetic variation. Given that these lines were derived through eight generations of artificial selection(1, 2), it is plausible that underlying genetic variation—potentially acting through methylation quantitative trait loci (meQTLs)—contributes to the observed epigenetic divergence(3-5). Alternatively, these signatures could represent stochastic epigenetic events stabilized through development. Future work integrating whole-genome sequencing with methylome analysis across generations will be necessary to resolve this question. Nonetheless, the stability and functional relevance of these pre-existing markers position them as valuable epigenetic biomarkers for resistance breeding, regardless of their genetic or epigenetic origins."

2. Clarity on 5mC vs. 5hmC contributions: The distinction between 5mC and 5hmC is a strength of the study. However, the conclusion that 5mC, not 5hmC, drives expression changes (Fig. 6) is based on grouped analyses. It would be helpful to include a more detailed discussion of why 5hmC may not correlate with expression in this context, especially given its known role in gene regulation in other systems. Could this be tissue-specific or related to the timing of infection?

Response: Thanks for your suggestion. We supplemented our discussion (Line 254-260).

"Notably, we also observed a positive correlation between genebody 5hmC levels and gene expression, suggesting that 5hmC may promote transcription. This finding is consistent with observations in the brain and embryonic stem cells, where 5hmC regulates transcription and cell fate(6–8). However, the effect of 5hmC on transcriptional output is much weaker than that of 5mC,

which is likely due to its transient nature(9). In contrast, 5mC exerts a stable and persistent regulatory effect in maintaining DNA methylation patterns"

3. Keywords optimization: Line 49. The current keywords ("DNA methylation; Viral susceptibility; Endocytosis; virus; DHAV-3") are appropriate but could be more specific for indexing purposes. Consider replacing "virus" with "Host-pathogen interaction" or "Epigenetics," and adding "Duck" or "Avian" to increase discoverability for readers interested in poultry or animal models.

Response: Thanks for your kind suggestion. We have revised it. (Line 49-50).

4. Results section organization: In section 2.4, the authors describe the correlation between 5mC/5hmC and gene expression, followed by subgroup analyses in section 2.5. The transition between these sections is smooth, but the logic could be further clarified by adding a brief introductory sentence at the beginning of section 2.5, such as: "Having established that genebody 5mC is the dominant regulatory layer, we next investigated the biological pathways associated with these methylation changes." This would help guide readers through the narrative.

Response: Thanks for your suggestion. We have added it. (Line 175-176).

5. Terminology consistency: Line 196-202. In the Results (section 2.4), the terms "genebody-high" and "genebody-low" are used, while in the Discussion, "intragenic methylation" is introduced. To avoid confusion, consider defining "intragenic methylation" as methylation within genebodies (including introns and exons) and using this term consistently throughout the manuscript, or at least clarifying the relationship between these terms when first introduced.

Response: Thank you for pointing this out. We have unified it in the manuscript as "genebody" for better understanding. (Line 29, Line 32, Line 255).

6. Figure 6 legend clarity: Line 204-211. In Fig. 6a and 6b, the four groups are labeled as "5mC-high/5hmC-high," "5mC-high/5hmC-low," etc. This is clear but lengthy. Consider defining these as "H/H," "H/L," "L/H," "L/L" in the figure with an explanatory footnote in the legend (e.g., "H: hypermethylated/hydroxymethylated; L: hypomethylated/hydroxymethylated") to reduce visual clutter.

Response: Thank you for your thoughtful suggestions. We have revised the group names in Fig. 6 and provided supplementary explanations in results (Line 159-163, 166-168, 170) and legends (Line 783-786).

"DMGs and DhMGs were classified as 5mC-hyper (hypermethylated-DMG), 5mC-hypo (hypomethylated-DMG), 5hmC-hyper (hypermethylated-DhMG), or 5hmC-hypo (hypomethylated-DhMG). Within the same 5hmC group (hyper/hypo), 5mC-hyper genes showed significantly lower log_2FC than 5mC-hypo genes at both time points (Fig. 6a, 6b)."

"At 0 hpi, G-hypo genes (hypomethylated-DMGs in genebody) showed higher log_2FC than G-hyper genes (hypermethylated-DMGs in genebody) within the same promoter group,"

"although promoter methylation showed some influence within the G-hyper group"

"Grouped gene expression changes of DMGs and DhMGs at 0 hpi (a) and 24 hpi (b). Grouped gene expression changes of DMGs in promoter and genebody regions at 0 hpi (c) and 24 hpi (d). hyper, hypermethylated. hypo, hypomethylated. P, promoter. G, genebody."

7. Gene name formatting: Line 270-271. In the text and figures, gene names (e.g., ACADM, CPT1A, PPARα) are correctly italicized. However, in some figure legends (e.g., Fig. 10b, 10c), gene names are not italicized. Please ensure consistent formatting throughout, following journal guidelines (typically italics for genes).

Response: Thank you for your reminder. We have revised it. (Line 805-807).

8. Discussion length: The Discussion is comprehensive but somewhat lengthy. Consider condensing the first paragraph, which broadly introduces virus-host interactions and DNA methylation, as much of this context is already covered in the Introduction. This would create more space to expand on the "double-edged sword" concept or the pre-existing signature origin as suggested in Major Comments.

Response: We thank the reviewer for this insightful observation. We have rewritten this paragraph. (Line 216-224). And we revised the discussion to provide a more detailed on the "double-edged sword" concept. (lines 306-317)

"DNA methylation is increasingly recognized as a key epigenetic regulator of host–pathogen interactions across diverse species, playing a conserved yet complex role in viral pathogenesis and host susceptibility(10–16). In this study, we employed integrated whole-genome bisulfite sequencing (WGBS), oxidative bisulfite sequencing (oxWGBS), and transcriptomics to dissect the DNA methylation landscapes underlying differential susceptibility to DHAV-3 in ducks. Our analysis reveals that an intragenic methylation signature, rather than promoter methylation, distinguishes resistant from susceptible ducklings and governs viral entry and downstream host responses, providing mechanistic insights into epigenetic regulation of antiviral defense.".

"The dual role of DNA methylation becomes evident when examining its pathway- and line-specific effects. In resistant ducklings, hypermethylation of endocytosis-related genes establishes a protective barrier at viral entry, while concurrent hypermethylation of fatty acid metabolism and autophagy pathways further reinforces resistance by limiting pro-viral host resources. In susceptible ducklings, however, hypomethylation of endocytosis genes renders host inherently permissive to viral entry—a clear pro-viral configuration. Paradoxically, despite this permissiveness, susceptible ducklings maintain hypermethylation of metabolic/autophagy pathways identical to resistant ducklings, suggesting an attempted host defense that is ultimately insufficient. Thus, DNA methylation functions as a context-dependent epigenetic checkpoint whose impact—anti- or pro-viral—is determined by the specific pathways affected and the host genetic background."

9. Conclusion section redundancy: The Conclusion largely restates key findings from the Discussion. While a summary is valuable, some sentences are nearly identical to those in the Discussion (e.g., "intragenic 5mC modifications, rather than promoter methylation, play a dominant role"). Consider shortening the Conclusion to 3-4 concise sentences that highlight the most novel take-home messages without repeating detailed results.

Response: We thank the reviewer for this insightful observation. We have rewritten the Conclusion. (Line 322-328)

"This study establishes that a pre-configured, intragenic DNA methylation landscape governs innate susceptibility to DHAV-3, primarily by setting the baseline for viral entry via endocytic pathway regulation. Beyond entry, this methylation program coordinates immune, metabolic, and autophagic responses, revealing a multi-layered epigenetic framework that shapes infection outcomes. These findings not only advance our understanding of host–pathogen epigenetic

interactions but also provide actionable targets for antiviral intervention and epigenetic marker-assisted breeding in poultry."

10. Methods section 5.6 cell culture medium composition: Line 461. The cell culture medium is described as "DMEM containing 10% FBS, 100 U/mL penicillin, and 0.1 mg/mL streptomycin." It would be helpful to specify whether this is high-glucose DMEM or another formulation, as hepatocyte viability can be affected by glucose concentration.

Response: Thank you for your reminder. Duck hepatocytes need to be cultured in a high-glucose DMEM environment. We have supplemented it in our method. (Line 414)

11. Methods section 5.7 drug treatment duration: Line 467. The text states that cells were treated with drugs for 6 h prior to infection, then infected and cultured for 1.5 h (5'AzaC) or 24 h (other drugs). It is unclear whether the drugs were present during the infection and post-infection culture period. Please clarify whether drugs were removed after pretreatment or maintained throughout the experiment.

Response: Thank you again for your reminder. We have complemented in methods. (Line 421)

"Drugs were halved with virus infection to maintain interference."

Reviewer 2

1. Lines 112-115: Fig. 1. The legends are not descriptive. Please provide the details of each panel of this figure including the statistical analyses, so that it becomes easier for reader to follow.

Response: Thank you for your detailed suggestions. We have supplemented the detailed information in Fig 1. (Line 743-751)

"(a) Representative gross liver morphology from uninfected (R0, S0) and DHAV-3-infected (R24, S24) ducklings at 24 hpi. (b) Representative hematoxylin and eosin (H&E) staining of liver sections from each group. (c) Relative mRNA expression levels of pro-inflammatory cytokines (IFN-γ, TNF-α, IL-6, IL-17A) in the liver at 0 hpi and 24 hpi. Expression levels were normalized to GAPDH and are presented as mean ± SEM (n = 3). (d) Viral loads in the liver, spleen, kidney, and bursa of Fabricius at 24 hpi. Viral RNA copies were quantified by qPCR and the DHAV-3 standard curve. Statistical significance was determined by two-tailed Wilcoxon rank-sum test.".

2. Line 118: 'DHAV-3 susceptibility' can be replaced with 'duckling's susceptibility to DHAV-3'. Besides, check this in Lines 215 and 324.

Response: Thank you for pointing this out. We have revised this description (Line 114, 178 and 262).

3. Lines 169-170: In Figure 4(e), it is difficult to differentiate between R0 vs S0 and R24 vs S24. Please use the appropriate symbols that offer better differentiation.

Response: Thank you for your reminder. We have adjusted the layout and colors of Figure 4 to present the DMRs and DhMRs between R0 vs S0 and R24 vs S24 more intuitively.

4. Lines 175-179: It is always preferred to provide the correlation coefficient and significant changes associated p-values within the manuscript text, as the readers will many a times find it difficult to follow these statistical analyses (by looking at the figure separately).

Response: Thanks for your kind suggestion. We have added the statistical information at the corresponding positions. (Line 150-156).

"both promoter and genebody regions at 0 hpi (promoter: p = 0.00015, r = -0.1827; genebody: p = 2.495 × 10-84, r = -0.5161) and 24 hpi (promoter: p = 8.283 × 10-46, r = -0.3214; genebody: p < 2.225 × 10-304, r = -0.5755) (Fig. 5a–5d). In contrast, promoter DhMGs showed no correlation with expression (0 hpi: p = 0.4217, r = 0.07764; 24 hpi: p = 0.2923, r = 0.0597), while genebody DhMGs exhibited a positive correlation—opposite to DMGs (0 hpi: p = 2.873 × 10-12, r = 0.2807; 24 hpi: p = 2.756 × 10-56, r = 0.3536)"

5. Line 181: Please check the p-value of Figure 5(c) genebody as it is extremely small.

Response: Thank you for pointing this out. We have double checked that the p-value is correct. We have added the statistical analysis method to the legend in Fig. 5 (Line 777).

"Statistical significance was determined by two-tailed Wilcoxon rank-sum test."

6. Lines 184-185: It is interesting to observe significant differences between the expression levels of controls (S0 vs R0) in the promoter region (although it is weaker). Have you investigated it?

Response: Thank you for your reminder. We have indeed investigated the promoter methylation differences between S0 and R0. In Fig. 5a and 5c, we observed a negative correlation between methylation and gene expression in promoter-anchored DMRs. We have added the following sentence in discussion. (Line 247-250).

"Although promoter methylation differences between resistant and susceptible ducklings were also observed, their effect sizes were substantially smaller than those associated with genebody methylation, consistent with the limited dynamic range of promoter methylation under steady-state conditions(17–19)"

7. Line 215: virus susceptibility or host susceptibility? Please check.

Response: Thank you for your reminder. We have made the correction for this (Line 178).

8. Lines 267-274: In Figure 10 (a, g, h, and i), legends on y-axis are not clear. Relative DHAV3 mRNA or Relative DHAV3 viral RNA copies? Please check. 'Con' is non-standard abbreviation of 'control'. It is better to write 'control'. In Figure 10 (b and c), please correct 'leve' on y-axis.

Response: Thank you for your careful review of our figure details. These data were generated by qPCR using the 2^(-ΔΔCt) method. We have standardized the y-axis labels across all panels in Figure 10 to "Relative DHAV3 viral RNA level.", and updated the method (Line 803-804, 808). And we have corrected the labels of the control group and the y-axis labels in Fig. 9b, 9c, and Fig.10.

"(a) Viral RNA levels of DHAV3 in the 5'Azac treated cells and control group at 24 hpi, measured by qPCR and normalized to GAPDH."

"Viral RNA levels of DHAV3 in cells treated with etomoxir…"

9. Line 362: I think Figure 11 can be re-structured to truly depict what is happening in your study. For instance, fatty acid metabolism and autophagy (hypermethylated in S-duckling) needs to be emphasized to differentiate between S- and R-ducklings.

Response: Thank you for your thoughtful suggestion regarding Figure 11. We have revised both Figure 11 and the discussion. (Line 306-317)

"The dual role of DNA methylation becomes evident when examining its pathway- and line-specific effects. In resistant ducklings, hypermethylation of endocytosis-related genes establishes a protective barrier at viral entry, while concurrent hypermethylation of fatty acid metabolism and autophagy pathways further reinforces resistance by limiting pro-viral host resources. In susceptible ducklings, however, hypomethylation of endocytosis genes renders host inherently permissive to viral entry—a clear pro-viral configuration. Paradoxically, despite this permissiveness, susceptible ducklings maintain hypermethylation of metabolic/autophagy pathways identical to resistant ducklings, suggesting an attempted host defense that is ultimately insufficient. Thus, DNA methylation functions as a context-dependent epigenetic checkpoint whose impact—protective or pro-viral—is determined by the specific pathways affected and the host genetic background."

10. Line 383: Please mention exactly from where the virus was procured. China or some other country?

Response: Thank you for your reminder. We have added it in the Methods (Line 332-335).

"The DHAV-3 strain (isolate 112803) was originally isolated from one-week-old ducklings during a duck viral hepatitis outbreak in China in 2011 and was provided by Key Laboratory of Animal Epidemiology of the Ministry of Agriculture, China."

11. Lines 386-387: Please inform the biological replicates taken by the authors to derive the conclusions as well experimental plan in the methods section to make it clearer to the readers.

Response: Thank you for your careful review of our experimental design. We have revised the description of this part (Line 337-340).

"For each experimental group, three biologically independent ducklings were used: uninfected resistant (R0, n = 3), uninfected susceptible (S0, n = 3), infected resistant (R24, n = 3), and infected susceptible (S24, n = 3). All samples were collected at 0 hpi (pre-infection) and 24 hpi."

12. Minor topographical corrections- Line 52, 265, 404.

Response: Thank you for your careful proofreading. We have corrected these in Line 53, 213 and 357.

"pathogens that hijack...", "pathways that are…", "Sequencing was performed…"

References

1. Liang S, Wang M-S, Zhang B, Feng Y, Tang J, Xie M, Huang W, Zhang Q, Zhang D, Hou S. 2021. NOD1 Is Associated With the Susceptibility of Pekin Duck Flock to Duck Hepatitis A Virus Genotype 3. Front Immunol 12:766740.
2. Wang X, Zhang J, Meng R, Jiang Y, Liang S, Zhang Y, Xie M, Zhou Z, Hou S. 2017. Host Differences Affecting Resistance and Susceptibility of the Second Generation of a Pekin Duck Flock to Duck Hepatitis A Virus Genotype 3. Front Microbiol 8:1128.
3. Hawe JS, Wilson R, Schmid KT, Zhou L, Lakshmanan LN, Lehne BC, Kühnel B, Scott WR, Wielscher M, Yew YW, Baumbach C, Lee DP, Marouli E, Bernard M, Pfeiffer L, Matías-García PR, Autio MI, Bourgeois S, Herder C, Karhunen V, Meitinger T, Prokisch H, Rathmann W, Roden M, Sebert S, Shin J, Strauch K, Zhang W, Tan WLW, Hauck SM, Merl-Pham J, Grallert H, Barbosa EGV, MuTHER Consortium, Illig T, Peters A, Paus T, Pausova Z, Deloukas P, Foo RSY, Jarvelin M-R, Kooner JS, Loh M, Heinig M, Gieger C, Waldenberger M, Chambers JC. 2022. Genetic variation influencing DNA methylation provides insights into molecular mechanisms regulating genomic function. Nat Genet 54:18–29.
4. Bk P, Nn V, Iv N, Cm N, M B. 2024. Methylome-wide and meQTL analysis helps to distinguish treatment response from non-response and pathogenesis markers in schizophrenia. Frontiers in psychiatry 15.
5. Fouéré C, Costes V, Hozé C, Raja Ravi Shankar A, Besnard F, Costa Monteiro Moreira G, Sorin V, Le Danvic C, Chaulot-Talmon A, Ali F, Deloche MC, Bonnet A, Sellem E, Jammes H, Fritz S, Boussaha M, Boichard D, Kiefer H, Sanchez M-P. 2025. Genetic regulation of sperm DNA methylation in cattle through meQTL mapping. BMC Genomics 26:771.
6. MacArthur IC, Dawlaty MM. 2021. TET Enzymes and 5-Hydroxymethylcytosine in Neural Progenitor Cell Biology and Neurodevelopment. Front Cell Dev Biol 9:645335.
7. Li H, Hu Z, Jiang H, Pu J, Selli I, Qiu J, Zhang B, Feng J. 2020. TET1 Deficiency Impairs Morphogen-free Differentiation of Human Embryonic Stem Cells to Neuroectoderm. Sci Rep 10:10343.
8. Ma L, Wang F, Li Y, Wang J, Chang Q, Du Y, Sadan J, Zhao Z, Fan G, Yao B, Chen J-F. 2023. Brain methylome remodeling selectively regulates neuronal activity genes linking to emotional behaviors in mice exposed to maternal immune activation. Nat Commun 14:7829.
9. Wu X, Zhang Y. 2017. TET-mediated active DNA demethylation: mechanism, function and beyond. Nat Rev Genet 18:517–534.
10. Jin J, Xu H, Wu R, Niu J, Li S. 2019. Aberrant DNA methylation profile of hepatitis B virus infection. J Med Virol 91:81–92.
11. Bernaudat F, Gustems M, Günther J, Oliva MF, Buschle A, Göbel C, Pagniez P, Lupo J, Signor L, Müller CW, Morand P, Sattler M, Hammerschmidt W, Petosa C. 2022. Structural basis of DNA methylation-dependent site selectivity of the Epstein-Barr virus lytic switch protein ZEBRA/Zta/BZLF1. Nucleic Acids Res 50:490–511.
12. Corley MJ, Pang APS, Dody K, Mudd PA, Patterson BK, Seethamraju H, Bram Y, Peluso MJ, Torres L, Iyer NS, Premeaux TA, Yeung ST, Chandar V, Borczuk A, Schwartz RE, Henrich TJ, Deeks SG, Sacha JB, Ndhlovu LC. 2021. Genome-wide DNA methylation

profiling of peripheral blood reveals an epigenetic signature associated with severe COVID-19. Journal of Leukocyte Biology 110:21–26.
13. Xiang X, Yu D, Li Z, Fros JJ, Wei J, Liu K, Li Z, Shao D, Li B, Kortekaas J, van Oers MM, Ma Z, Pijlman GP, Qiu Y. 2024. Japanese encephalitis virus-induced DNA methylation contributes to blood-brain barrier permeability by modulating tight junction protein expression. J Neuroinflammation 21:277.
14. Crimi E, Benincasa G, Figueroa-Marrero N, Galdiero M, Napoli C. 2020. Epigenetic susceptibility to severe respiratory viral infections and its therapeutic implications: a narrative review. Br J Anaesth 125:1002–1017.
15. He L, Liang X, Wang Q, Yang C, Li Y, Liao L, Zhu Z, Wang Y. 2022. Genome-wide DNA methylation reveals potential epigenetic mechanism of age-dependent viral susceptibility in grass carp. Immun Ageing 19:28.
16. Yadav RK, Chattopadhyay D. 2011. Enhanced viral intergenic region-specific short interfering RNA accumulation and DNA methylation correlates with resistance against a geminivirus. Mol Plant Microbe Interact 24:1189–1197.
17. Yamazaki J, Matsumoto Y, Jelinek J, Ishizaki T, Maeda S, Watanabe K, Ishihara G, Yamagishi J, Takiguchi M. 2021. DNA methylation landscape of 16 canine somatic tissues by methylation-sensitive restriction enzyme-based next generation sequencing. Sci Rep 11:10005.
18. Dayeh T, Volkov P, Salö S, Hall E, Nilsson E, Olsson AH, Kirkpatrick CL, Wollheim CB, Eliasson L, Rönn T, Bacos K, Ling C. 2014. Genome-wide DNA methylation analysis of human pancreatic islets from type 2 diabetic and non-diabetic donors identifies candidate genes that influence insulin secretion. PLoS Genet 10:e1004160.
19. Du X, Han L, Guo A-Y, Zhao Z. 2012. Features of methylation and gene expression in the promoter-associated CpG islands using human methylome data. Comp Funct Genomics 2012:598987.

Re: Spectrum00565-26R1 (**DNA Methylation-Mediated Suppression of Endocytosis Confers Resistance to Duck Hepatitis A Virus Type 3**)

Dear Prof. Xia Wang:

Your manuscript has been accepted, and I am forwarding it to the ASM production staff for publication. Your paper will first be checked to make sure all elements meet the technical requirements. ASM staff will contact you if anything needs to be revised before copyediting and production can begin. Otherwise, you will be notified when your proofs are ready to be viewed.

Data Availability: ASM policy requires that data be available to the public upon online posting of the article, so please verify all links to sequence records, if present, and make sure that each number retrieves the full record of the data. If a new accession number is not linked or a link is broken, provide production staff with the correct URL for the record. If the accession numbers for new data are not publicly accessible before the expected online posting of the article, publication may be delayed; please contact ASM production staff immediately with the expected release date.

Publication Fees: For information on publication fees and which article types have charges, please visit our website. We have partnered with Copyright Clearance Center (CCC) to collect author charges. If fees apply to your paper, you will receive a message from no-reply@copyright.com with further instructions. For questions related to paying charges through RightsLink, please contact CCC at ASM_Support@copyright.com or toll free at +1-877-622-5543. CCC makes every attempt to respond to all emails within 24 hours.

ASM Membership: Corresponding authors may join or renew ASM membership to obtain discounts on publication fees. Need to upgrade your membership level? Please contact Customer Service at Service@asmusa.org.

Embargo Policy: A press release may be issued as soon as the manuscript is posted on the Spectrum Latest Articles webpage. The corresponding author will receive an email with the subject line "ASM Journals Author Services Notification" when the article is available online.

The ASM Journals program strives for constant improvement in our submission and publication process. Please tell us how we can improve your experience by taking this quick Author Survey.

Thank you for submitting your paper to Spectrum.

Sincerely,
Zsolt Toth
Editor
Microbiology Spectrum
